# Pretreatment of Plastic Waste: Removal of Colorants from HDPE Using Biosolvents

**DOI:** 10.3390/molecules27010098

**Published:** 2021-12-24

**Authors:** Ana M. Ferreira, Isa Sucena, Vanessa Otero, Eva Mariasole Angelin, Maria João Melo, João A. P. Coutinho

**Affiliations:** 1 CICECO—Aveiro Institute of Materials, Department of Chemistry, University of Aveiro, 3810-193 Aveiro, Portugal; isasucena@ua.pt (I.S.); jcoutinho@ua.pt (J.A.P.C.); 2 LAQV-REQUIMTE, Department of Conservation and Restoration, Faculdade de Ciências e Tecnologia, Universidade NOVA de Lisboa, 2829-516 Caparica, Portugal; van_otero@fct.unl.pt (V.O.); e.angelin@campus.fct.unl.pt (E.M.A.); mjm@fct.unl.pt (M.J.M.); 3 VICARTE, Department of Conservation and Restoration, Faculdade de Ciências e Tecnologia, Universidade NOVA de Lisboa, 2829-516 Caparica, Portugal; 4 Conservation Science Department, Deutsches Museum, Museumsinsel 1, 80538 Munich, Germany

**Keywords:** high-density polyethylene, closed-loop recycling, solvent extraction, dissolution-precipitation, additives, pigments, dyes, circular economy

## Abstract

Plastics recycling remains a challenge due to the relatively low quality of the recycled material, since most of the developed recycling processes cannot deal with the additives present in the plastic matrix, so the recycled products end up in lower-grade applications. The application of volatile organic solvents for additives removal is the preferred choice. In this study, pretreatment of plastic packaging waste to remove additives using biosolvents was investigated. The plastic waste used was high-density polyethylene (HDPE) with blue and orange colorants (pigment and/or dye). The first step was to identify the type of colorants present in the HDPE, and we found that both plastics presented only one colorant that was actually a pigment. Then, limonene, a renewable solvent, was used to solubilize HDPE. After HDPE dissolution, a wide range of alcohols (mono-, di-, and tri-alcohols) was evaluated as antisolvents in order to selectively precipitate the polymer and maximize its purity. The use of limonene as solvent for plastic dissolution, in combination with poly-alcohols with an intermediate alkyl chain length and a large number of hydroxyl (OH) groups, was found to work best as an antisolvent (1,2,3-propanetriol and 1,2,4-butanetriol), leading to a removal of up to 94% and 100% of the blue and orange pigments, respectively. Finally, three cycles of extraction were carried out, proving the capability of the solvent and antisolvent to be recovered and reused, ensuring the economic viability and sustainability of the process. This pretreatment provides a secondary source of raw materials and revenue for the recycling process, which may lead to an increase in the quality of recycled polymers, contributing to the development of an economical and sustainable recycling process.

## 1. Introduction

Plastics (polymers) are widespread in most activities and our daily lives. In 2018, the global polymer production reached 360 Mt, which is anticipated to rise four-fold by 2050 [1]. Of this total, 61.8 Mt was consumed in the European Union (EU), with packaging being the most significant use, representing 40% of this value [2]. However, plastics have a short life cycle, generating large amounts of waste with high prevalence in the environment due to their lower biodegradability, becoming one of the imminent environmental concerns of the 21st century. The European Commission is encouraging plastic waste recycling through the implementation of the Circular Economy Package, aiming to recycle 50% of the plastic waste generated in the EU by 2030 [3,4], to achieve the targets proposed by the United Nations 2030 Sustainable Development Goals (SDGs).

The most common approach for plastic recovery is mechanical recycling [5]. However, this recycling process produces recycled plastic of inferior quality [6] that can only be used in low-value applications, thus not suppressing the need for virgin feedstock. New techniques are emerging to solve this problem, such as plastics depolymerization, either by using enzymes [7] or chemicals [8]. Depolymerization is an interesting approach since the polymer is degraded into monomers that can be used to remake virgin-grade material, while preserving the initial material properties [6]. Using (bio)catalysis to convert plastic waste into circular material streams is distinctly different from converting plastics into fuels or using them for energy recovery [9]. Thus, this method has the advantage of ensuring that the material value is retained within the plastic economy indefinitely, contributing to closed-loop recycling [10]. However, the presence of additives in plastics can pose a problem for depolymerization, since they can affect the enzyme’s activity [7] or lead to a feedstock that needs an extensive purification process [8]. Therefore, additives (stabilizers, flame retardants, colorants, plasticizers, etc.) used to improve the performance of polymers are one of the major bottlenecks in the closed-loop recycling of plastic, i.e., obtaining a high-quality recycled polymer. Thus, it is essential to remove additives before the recycling process to obtain high quality recycled polymers [11]. So, the development of more sustainable strategies in the production of high-quality raw material from plastic waste is crucial.

Presently, solvent extraction (SE) techniques, such as solid-liquid extraction (SLE) and dissolution-precipitation, are the most promising methods for removing additives from plastics [3]. Compared to other approaches, the major advantages of SE are the simplicity of the equipment and operation for separation, and the high purity of the recycled plastic obtained [3,6,11]. Moreover, SE presents the significant advantage of yielding a plastic with the same quality as the virgin material [6]. One type of additive that has a marked impact on plastics recycling is the colorants, since color in recycled material reduces its commercial value; thus, its removal is essential. Colorants, due to their physical behavior, are classified into two types: dyes and pigments. Dyes are organic compounds, and they are soluble in a polymer matrix and produce a transparent look [12]. Pigments can be organic or inorganic and are insoluble in the polymer matrix [12]. If the pigments have a size smaller than 0.2 mm, they produce a transparent quality in the polymer matrix, but if the particles are bigger than 0.2 mm, the plastic appears opaque [12]. Colorants have been extracted from polymers using traditional technologies, such as Soxhlet [13], as well as innovative techniques such as microwave assisted extraction (MAE) [13,14] and supercritical fluid extraction (SFE) [13]. Jiménez et al. [13] compared the use of three different SLE techniques (Soxhlet, MAE, and SFE) for the removal of three azo dyes (Sudan I, Sudan IV, and disperse red 1) commonly found in toys. Their results demonstrated that the dyes’ extraction, performed with the most innovative techniques (MAE and SFE) were more attractive, since these techniques present a shorter extraction time and are much more efficient than Soxhlet extraction in the removal of dye from poly(vinyl chloride) [13]. According to the authors, MAE is the technique that removed higher percentages of dye: 99.4% for Sudan I, 59.5% for Sudan IV, and 99.8% for disperse red 1, using methanol at 120 °C for 20 min of extraction and a solid–liquid ratio (S/L ratio) of 0.03 [13]. Noguerol-Cal et al. [14] also showed that MAE is a promising technique for removing the colorants Sudan IV, Dimethyl Yellow, and Solvent Blue 35 from plastic matrices. More specifically, the authors were able to achieve 100% removal of azo dyes from polypropylene using MAE and dichloromethane as the extraction solvent [14]. Another SE approach that has been applied to remove colorants from plastics is dissolution-precipitation [15,16]. Arends et al. [15] removed inorganic color pigments (80% of titanium dioxide and iron (III) oxide, and 90% of chromium (III) oxide) from acrylonitrile butadiene styrene (ABS) by dissolution of the polymer in CreaSolv^®^SB and its precipitation by CreaSolv^®^SBF, but it is not known what kind of solvents the authors applied to obtain these results. Another study demonstrated the removal of titanium dioxide (white inorganic pigment) from polyethylene (PE) samples using the o-dichlorobenzene/o-xylene solvent system, but the authors could only remove 15% of the colorant [16]. Most solvents that have been used in the extraction of colorants from polymers (as well as other additives) are volatile organic compounds, often chlorinated [13,14,16]. Recently, some of these solvents have been restricted or subject to authorization by REACH, such as n-hexane, methanol, and toluene, all of which are used in solvent extraction for polymer recycling [6]. Thus, there is a need to develop more environmentally friendly and cost-effective pretreatments to remove and recover the additives from plastic waste.

Renewable solvents, such as the biosolvents cyrene, gamma valerolactone, limonene, glycerol ethers, to name a few, have emerged as interesting options in different applications [17]. Biosolvents are biobased solvents (derived from natural products such as cellulosic waste or biomass) of renewable origin. They are biodegradable and less toxic than most organic solvents. Despite their remarkable potential to minimize the environmental impacts associated with the recycling process and, consequently, to enable sustainable additive extraction/removal, these green solvents have seldom been investigated as solvents for the extraction of additives, especially colorants, from plastic waste. To the best of our knowledge, there is only one study in which the authors used biosolvents, specifically butanediol, to remove the inorganic pigment cadmium sulfate from high-density polyethylene (HDPE) [18]. 

In this work, we aimed to develop a sustainable pretreatment to remove and recover additives, specifically colorants, from plastic packaging waste by SE using biosolvents, in order to facilitate the recycling process by depolymerization. These studies were carried out using HDPE, one of the top four representatives in plastic packaging waste [19]. We used HDPE plastic packaging with blue and orange different colorants in this study. First, we identified the type of colorant (dye/pigment) present in each HDPE. Second, a pretreatment to remove the colorants from HDPE was applied using the dissolution-precipitation technique. Third, the most promising (bio)solvents for the dissolution of plastics were selected, with toluene being adopted as the benchmark. Alcohols (mono-, di-, and tri-alcohols) were used to induce the precipitation of HDPE and to verify the effect of its structure on the percentage removal of the colorants, i.e., the alkyl chain length, the number of hydroxyl (OH) groups, and the distance between the OH groups in the alkyl chain. Fourth, the recovered polymer was characterized to evaluate if its thermal properties were maintained after the pretreatment. Fifth, the crystallinity of the recovered HDPE was also evaluated, since it has a direct impact on the mechanical properties of the polymer, such as yield stress, modulus of elasticity and impact strength [9,20]. Finally, the recyclability of the most promising solvents system (solvent:antisolvent) was studied.

## 2. Results and Discussion

### 2.1. Colorants Identification

**Figure 1:**
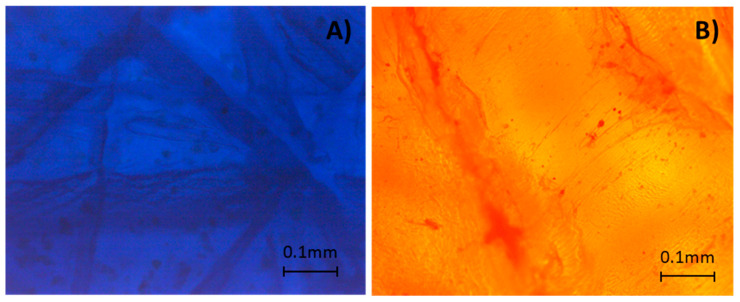
Microscopy images of (**A**) blue HDPE and (**B**) orange HDPE under reflected visible light (10× magnification, dark field).

First, both HDPE samples were analyzed by MO under a magnification of 10× and a darkened illumination. The microscopic images in Figure 1 show the existence of blue or orange particles/aggregates dispersed in the matrix. Therefore, we concluded that both samples had pigments as coloring agents, since they were dispersed in the matrix and not dissolved. Furthermore, since the background color of the microscopic images was constant for both polymeric matrices, both had only one pigment in their composition.

Regarding the chemical composition of the pigments, we supposed, based on the literature, that blue HDPE colorant should be the blue organic pigment from the family of phthalocyanines, named copper phthalocyanine blue (PB15), whereas orange HDPE should be the organic orange pigment from the benzimidazolone pigment family, named cromophtal orange (PO64). These considerations are based on the fact that these pigments are the most economical among the usual blue and orange pigments in the polyolefins [21,22,23]. The analysis of the blue HDPE by μ-EDXRF (Figure S1) showed the presence of copper, which suggested the presence of PB15. This hypothesis was unequivocally confirmed by μ-Raman analysis identifying PB15 (Figure S2). Moreover, the presence of other inorganic additives containing calcium and titanium in the blue HDPE was observed by μ-EDXRF (Figure S1). Conversely, the analysis of the orange HDPE by μ-EDXRF (Figure S1) demonstrated the presence of calcium, and μ-Raman identified the synthetic organic pigment PO64 [24] as the main coloring agent. The identification of PB15 and PO64 is supported by the literature [25,26].

### 2.2. Pretreatment of HDPE

The pretreatment (dissolution-precipitation) was developed in order to find a solvent:antisolvent system based on renewable solvents (biosolvents) with a high capacity to dissolve HDPE and to precipitate it selectively, i.e., to obtain a polymer free of colorant (pigment).

#### 2.2.1. Dissolution

Regarding HDPE dissolution, the alternative solvents selected were cyclohexane and D-limonene. These solvents were chosen from a solubility simulation program based on Hansen’s three-dimensional method [27], where solvents having the three solubility parameters most similar to those of HDPE were chosen. Moreover, the solvents traditionally used for dissolution of HDPE are toluene [28] and xylene [23], so these solvents were also used for comparison purposes. Table 1 summarizes the operating conditions and the results obtained for the HDPE dissolution for the different solvents in this study. The dissolution temperatures of toluene and xylene were selected according to the literature [23,28] (110 and 140 °C, respectively, as the boiling temperatures of the solvents); so, the dissolution temperature of cyclohexane was also its boiling temperature (80 °C). However, D-limonene has a very high boiling temperature, so we decided to use a dissolution temperature of 140 °C, which was the maximum temperature in this study amongst the considered solvents. Furthermore, a longer dissolution time was used for the cyclohexane to ensure that the time was not the limiting factor for the dissolution of the HDPE. The kinetic control of dissolution is one of the factors having more of an impact on the dissolution process, being more relevant for macromolecules such as HDPE than for low-molecular-weight compounds (where mixing is easier and molecular diffusion is faster) [29]. 

Table 1 shows that the dissolution of the polymeric matrices did not occur with the cyclohexane solvent, so cycloalkanes do not seem to be a suitable option for the dissolution of HDPE. On the other hand, the solvents toluene, xylene, and limonene were able to dissolve the polymeric matrices. Thus, the aromatic hydrocarbons (toluene and xylene) demonstrated the ability to dissolve HDPE, which would be expected considering the results in the literature [23,28]. Similarly, but despite not having aromaticity, limonene also showed the ability to dissolve HDPE, which is in agreement with several studies demonstrating that this biosolvent has the potential to replace toluene in many processes [30]. Moreover, when toluene and limonene were used as solvents for dissolving HDPE, there were no visible changes in the color of the polymer after dissolution, whereas in the case of xylene, a change occurred in the color from blue to a purple/violet. 

In summary, limonene was selected as alternative solvent for polymer dissolution because it is a renewable biosolvent and presents low toxicity [31] compared to other solvents such as toluene. Moreover, toluene was also adopted for comparison purposes because it is the organic solvent traditionally used in this process [28]. Before the precipitation step, the dissolution capacity of limonene was evaluated at the toluene dissolution conditions (T = 110 °C, 700 rpm for 30 min), since it would be more advantageous to apply the same operating conditions for both solvents. The results showed that the ability of limonene to dissolve HDPE at toluene’s operating conditions was maintained. 

#### 2.2.2. Precipitation

A wide range of alcohols from mono-, di-, and tri-alcohols (20 in total) was used as antisolvents to precipitate HDPE. More specifically, we intended the solubility of HDPE in the solution to gradually decrease with the addition of the alcohol (antisolvent), and consequently, the interaction between the solvent and HDPE would decrease, while the interaction between the HDPE chains would strengthen, leading to the precipitation of the polymer. Alcohols were chosen as antisolvents considering environmental and economic factors, since they are mainly derived from renewable sources and have lower toxicities than volatile organic solvents [32,33]. In addition, the choice of alcohols as antisolvents was also based on previous results in the literature, since the efficiency of 2-propanol [34] and methanol [23] as antisolvents for LDPE precipitation was demonstrated, increasing the probability that alcohols are a suitable option as antisolvents for HDPE precipitation. 

In order to maximize the yield of the precipitated polymer, three solvent:antisolvent ratios were initially evaluated, i.e., 1:residual, 1:1, and 1:3, in order to verify the most efficient to precipitate the polymer. The precipitation of the polymer started to be visible from the 1:1 ratio, but the 1:3 ratio was the best ratio for both dissolution solvents (toluene and limonene), as shown in Table S1. The results presented in Table S1 are related to the use of the obtained 1,2-ethanediol as the antisolvent, but the remaining antisolvents presented similar results. Thus, the following studies were conducted using a solvent:antisolvent ratio of 1:3.

**Figure 2:**
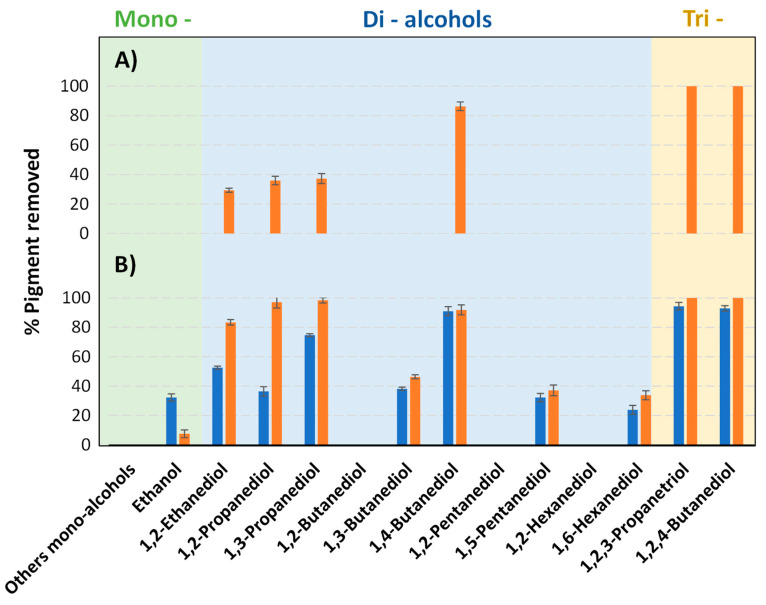
Percentage of pigment removed for (■) blue HDPE and for (■) orange HDPE. Polymer matrix dissolved in (**A**) toluene or (**B**) limonene using a solvent:antisolvent ratio of 1:3.

Afterward, the selectivity of the polymer precipitation was determined by the percentage of pigment removed by FT-Raman spectroscopy, as explained in Section 3.3.2. Figure 2 (Table S2) shows the percentage of pigment removed, for each solvent:antisolvent system. Overall, the results showed that the removal of the pigments from the polymer was more efficient when limonene was used as solvent (Figure 2B) to dissolve the polymers (Figure 2A), since a higher number of alcohols was able to remove the pigments partially or completely from the HDPE samples dissolved in limonene. Furthermore, for the samples dissolved in toluene, only the pigment present in the orange HDPE could be removed, with the tri-alcohols (1,2,3-propanetriol and 1,2,4-butanetriol) leading to the best results: 100% of pigment removed. When limonene was used to dissolve the polymer samples, it was possible to extract both pigments from HDPE samples. The most successful antisolvents were 1,2,3-propanetriol and 1,2,4-butanetriol (tri-alcohols) ere, removing up to 94% of the blue pigment and 100% of the orange pigment, respectively.

**Figure 3:**
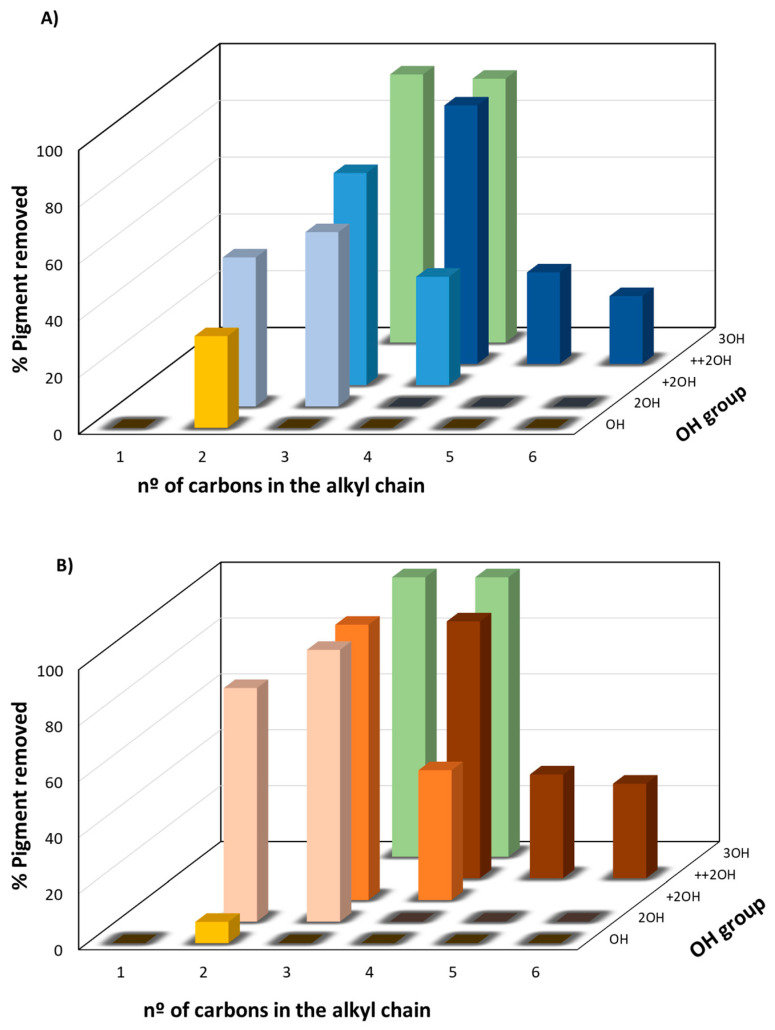
Influence of the chemical structure of the antisolvent (alcohol), on the removal percentage of (**A**) blue and (**B**) orange pigment from HDPE, using limonene as the solvent. (■) mono-alcohols (OH), (■, ■) di-alcohols with OH groups with short distance (2OH), (■, ■) di-alcohols with OH groups with middle distance (+2 OH), (■, ■) di-alcohols with OH groups with long distance (++2 OH), and (■) tri-alcohols (3 OH).

A more detailed analysis was then performed regarding the influence of the chemical structure of the alcohol (antisolvent) on the percentage of pigment removal from HDPE samples using limonene as the solvent (Figure 3). These data were chosen as limonene was the most promising solvent for recovering HDPE without the presence of colorants. This analysis considered three main aspects: (i) number of OH groups present in the alcohol (one to three OH groups), (ii) the size of the alkyl chain (one to six carbons), and (iii) the distance between OH groups in the alkyl chain of the di-alcohols (short distance: OH groups on carbons next to each other; middle distance: OH groups on the initial carbon and in the middle of the alkyl chain.; long distance: OH groups on the initial and final carbon of the alkyl chain).

**Figure 4:**
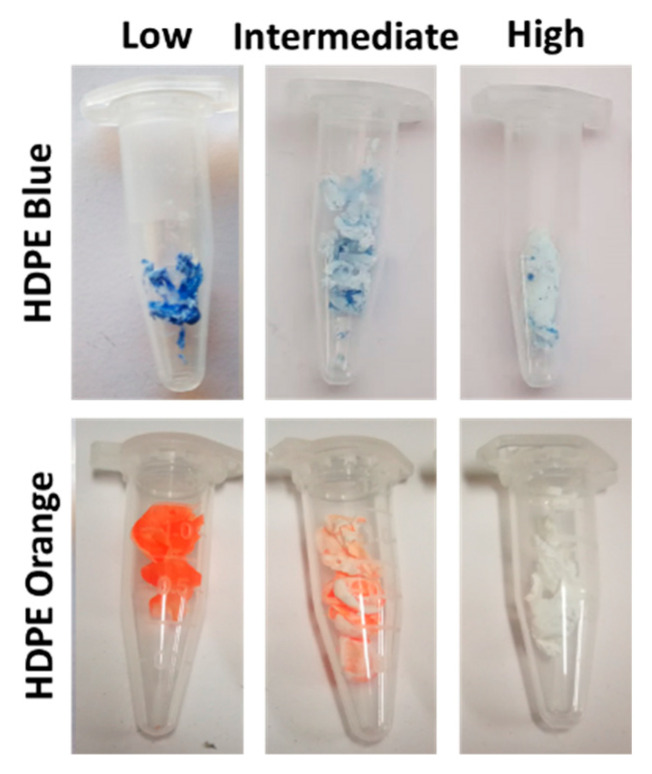
Summary of the visual results obtained after the precipitation process of the polymer samples dissolved using limonene as the solvent. The results obtained were divided into 3 levels of pigment removal from HDPE: low, <20%; intermediate, between 20 to 80%; high, >80%.

Figure 3 shows that for both pigments, the increase in the number of OH groups in the alcohol (mono-, di-, and tri-alcohols), i.e., the polarity increase, favored their removal. Concerning the increase in the alkyl chain (one to six carbons), i.e., the addition of carbons to the alcohol main chain, up to a four-carbon alkyl chain, led to an increase in the percentage of pigment removal, being more visible for di-alcohols (Figure 3). Conversely, a decrease in the percentage of pigment removed was observed for alcohols with an alkyl chain with more than four carbons (i.e., pentanediol and hexanediol), because the increase in the alkyl chain length led to a decrease in the polarity of the alcohols, which opposes the polarity effect resulting from the rise in the number of OH groups (Figure 3), and consequently decreases the ability of the alcohols to selectively precipitate the polymer [35]. Thus, it is necessary to have a balance between the number of OH groups and the number of carbons of the alkyl chain present in the alcohol, and the best combination, considering the results obtained was: three OH groups and alkyl chains with three to four carbons (1,2,3-propanetriol and 1,2,4-butanetriol), with a removal percentage up to 94% for the blue pigment and 100% for the orange pigment. In addition, the effect of the distance of the OH groups along the alkyl chain was studied for the di-alcohols. For the 1,2-butanediol, 1,3-butanediol, and 1,4-butanediol, we observed that with an increase in the OH groups’ distance, the percentage of pigment removed increased from 0% to 38% to 91% for the blue pigment and from 0% to 46% to 92% for the orange pigment, respectively (Figure 3). For propanediol, pentanediol, and hexanediol, the same tendency was also observed, with the increase in the OH groups’ distance, the percentage of pigment removed increased. Thus, a balance between the number of OH groups, the number of carbons in the alkyl chain, and the distance between the OH groups is necessary to obtain selective precipitation of the HDPE and remove a high percentage of the pigments. As an illustration of these results, three samples with different amounts of pigment removed are presented in Figure 4.

To the best of our knowledge, our report is the first in the experimental application of the dissolution-precipitation technique for the extraction of colorants from HDPE based on the use of biosolvents. We found that the removal of pigments was more efficient when using limonene (instead of the usual volatile organic solvent toluene) as the solvent for the plastic dissolution; poly-alcohols with an intermediate alkyl chain length and a high number of hydroxyl (OH) groups were found to work best as the antisolvent (1,2,3-propanetriol and 1,2,4-butanetriol). The results obtained here show the potential of these biosolvents for removing additives from plastic waste, facilitating full depolymerization, which could lead to higher quality of recycled polymers and to sustainable closed-loop plastic recycling.

Finally, the HDPE samples obtained with the best system and respective pigments were analyzed by μ-EDXRF and μ-Raman, i.e., the blue and orange HDPE samples after pretreatment with the limonene:1,2,3-propanetriol system. The use of these techniques, especially μ-Raman, is related to the fact that Raman spectroscopy (such as μ-Raman) is a useful technique for the identification of polymers [36,37] and their additives [38,39], because it allows the discrimination of plastic and additives (minimizing the occurrence of false positives), is a nondestructive technique, and requires small amounts of sample. In addition, Raman spectroscopy is a sensitive technique, allows the understanding of changes in the molecular structure level of PE, and distinguishes low-density polyethylene (LDPE) from high-density polyethylene (HDPE). The analyses of the recovered HDPE by μ-EDXRF and μ-Raman confirmed that the recovered polymer from the orange HDPE sample after pretreatment was completely free of pigment (Figures S3 and S4). Additionally, we observed that the other additives present in the orange HDPE before extraction (containing iron and calcium) were also removed with this pretreatment, highlighting the potential of the developed process. Furthermore, μ-Raman analysis of the recovered samples from the blue and orange HDPE (Figure S4) confirmed that the blue pigment extracted was PB15 and the orange pigment extracted was PO64, and showed a minor contamination with HPDE for the pigments recovered (ca. 2–5% (*w*/*w*)).

### 2.3. Chemical Characterization of the Polymer before and after Pretreatment

#### 2.3.1. Fourier Transform Infrared Spectroscopy (FT-IR)

**Figure 5:**
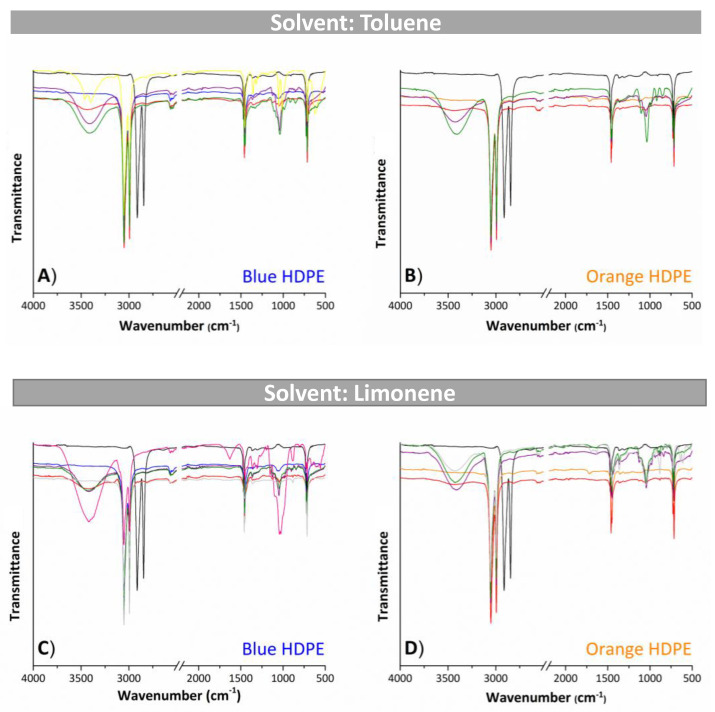
Vibrational spectra obtained by FT-IR of recovered HDPE samples under the pretreatment conditions. (**A**) Blue HDPE and (**B**) orange HDPE, both treated with toluene as solvent, and (**C**) blue HDPE and (**D**) orange HDPE, both treated with limonene as solvent. The different spectra represent the recovered HDPE using the following antisolvents: (━) methanol, (━) 1,2-ethanediol, (━) 1,2-propanediol, (━) 1,4- butanediol, (━) 1,6-hexanediol, (━) 1,2,3-propanetriol, and (━) 1,2,4-butanetriol. Controls: (━) pure HDPE, (━) blue HDPE, and (━) orange HDPE.

After the pretreatment, the samples of recovered HDPE were characterized by FT-IR, as presented in Figure 5, to identify possible structural changes. The spectra of the samples were grouped by solvent for each type of HDPE, i.e., one spectrum of a mono-alcohol (methanol), a di-alcohol (1,4-butanediol), and a tri-alcohol (1,2,3-propanetriol) was plotted to represent each alcohol type, and all spectra showing relevant changes were also plotted. The spectra of all recovered samples are shown in Figures S5–S8. In addition, Tables S3–S6 report a compilation of characteristic bands present in the polymer samples for each studied solvent:antisolvent system to facilitate interpretation of the spectra.

The characteristic bands of HDPE at 2912 and 2847 cm^−1^ were assigned to CH stretching in the −CH_2_- groups; at 1463cm^−1^, there was a CH bending band of the CH_2_ groups, while the band at 1375 cm^−1^ was assigned to the CH_3_ group; and at 720 cm^−1^, the band was characteristic of the CH_2_ group for n ≥ 4 (associated with rocking motion) [23,40,41] (Figure S9). All the peaks of recovered HDPE were identical to those of pure HDPE (Figure 5 and Figures S5–S8), indicating that HDPE remained stable during recycling. Furthermore, most spectra exhibited bands related to the presence of pure alcohols: a large stretching absorption band in the range of 3400–3300 cm^−1^, characteristic of the OH group; a stretching band from 1260 to 1000 cm^−1^, characteristic of the CO group (where primary alcohol: 1050 cm^−1^, secondary alcohol: 1100 cm^−1^; tertiary alcohol: 1150 cm^−1^) [42]. These results indicated that the alcohols used in the dissolution-precipitation process as antisolvents were not completely removed from the recovered HDPE sample, and the intensity of these bands depended on the amount of antisolvent (alcohol) present in the polymer matrix. The presence of the alcohols on the recovered polymer is also visible on the TGA presented below. Note that the presence of the alcohol did not alter the chemical structure of the polymer, since a sharp peak between 3650 and 3600 cm^−1^, characteristic of the free OH group that appears with the dilution of the alcohol in another compound, did not appear, so alcohols were only retained in the polymer matrix of the recovered HDPE samples [23].

#### 2.3.2. Thermogravimetric Analysis (TGA)

TGA curves were determined for the recovered HDPE with more than 80% pigment removed and compared to the reference samples (pure HDPE, blue HDPE, and orange HDPE) to evaluate the pretreatment effect on the thermal properties of the polymer. From the results of the thermogravimetric curves (Figure S10 and Table S7), the reference samples (pure HDPE, blue HDPE, and orange HDPE) were degraded in a single-step process between room temperature and 600 °C, whereas the recovered HDPE samples showed mass losses in the same temperature range. From the results of the thermogravimetric curves, we analyzed the corresponding temperatures for the maximum decomposition rate (T_max_) of the HDPE and the temperature at which 1% of HDPE decomposed (T_onset_).

The first mass loss observed in the recovered samples is related to the evaporation of the alcohol (antisolvent) that was retained in the HDPE matrix after the dissolution-precipitation process, as discussed above. The second mass loss corresponds to the decomposition of the HDPE. According to the results obtained, the decomposition temperature values for blue HDPE (T_onset_ = 451.9 °C and T_max_ = 479.4 °C) and orange HDPE (T_onset_ = 447.0 °C and T_max_ = 475.0 °C) were only marginally different from the values obtained for pure HDPE (T_onset_ = 454.0 °C and T_max_ = 479.1 °C, which are in agreement with the values in the literature [43,44]). These differences in decomposition temperature values may be related to the presence of additives in the plastics, which may have slightly altered the structure of the polymer and, consequently, its decomposition temperature values. 

These results support the conclusions obtained by FT-IR (Section 2.3.1), indicating that the alcohol is only retained in the polymer matrix; otherwise, the T_max_ and T_onset_ values of the recovered HDPE samples would have shifted more significantly in relation to the reference values. Thus, the results obtained suggested that the thermal properties of the recovered HDPE samples, in this case, decomposition temperature after the dissolution-precipitation process, did not change significantly compared to the HPDE source.

#### 2.3.3. Differential Scanning Calorimetry (DSC)

DSC was performed to study the melting behavior and crystallinity of the recovered HDPE samples with at least 80% pigment removed compared to the reference samples (pure HDPE, blue HDPE, and orange HDPE). DSC was performed to study the fusion behavior and crystallinity of the recovered HDPE samples. Notably, the cooling cycle was not performed because the DSC analyses were performed together with the TGA. The melting temperature (T_m_), the heat of fusion (∆H_f_), and the degree of crystallinity (DC) obtained for the recovered samples are summarized in Table 2 (Figure S11).

The melting temperature (T_m_) of the recovered samples remained almost unchanged compared with pure HDPE (140.1 °C), blue HDPE (140.0 °C), and orange HDPE (138.9 °C), with a variation up to 4.5% in relation to the respective HDPE source, which is not a significant variation, since the T_m_ of HDPE can vary from 130 to 140 °C. 

The degree of crystallinity (DC) was calculated according to: DC = (ΔH_f_/ΔH_f_*) × 100%, where ΔH_f_ is the actual heat of fusion of the HDPE sample tested, obtained by measuring the area under its thermogram peak; ΔH_f_* is equal to 293 J/g, corresponding to the heat of fusion of perfectly crystalline HDPE [45,46]. The pure HDPE, blue HDPE, and orange HDPE had a degree of crystallinity of 85.2%, 85.1%, and 75.5%, respectively. The recovered HDPE had a crystallinity degree ranging from 66.4–94.4% (Table 2). Thus, the crystallinity values of the recovered HDPE in some cases increased a little in relation to the HDPE source, but, in other cases, the crystallinity values decreased. This variation in crystallinity can be justified because it is a kinetic phenomenon. More specifically, after the HDPE dissolution process at a temperature of 110 °C, its precipitation was induced by adding a cold antisolvent. However, disordered (amorphous) polymer chains need time to organize themselves into ordered (crystalline) regions, but, in this case, it may have caused a too-sudden cooling, and the chains did not have enough time to reorganize, thus affecting the crystallization of the recovered HDPE samples. Furthermore, this variation in crystallinity may also have been related to the plasticization of HDPE due to the presence of residual amounts of the antisolvent (alcohol) retained in the polymer matrix [47].

### 2.4. Recyclability of Solvents

After identifying the most promising solvent:antisolvent system for removing the pigments present in HDPE, the recyclability of the solvent system was studied to maximize the relationship between the cost, efficiency, and sustainability of the process. The system chosen was one of the most promising, i.e., limonene:1,2,3-propanetriol, to remove the orange pigment from HDPE. This choice was based on the cost of the antisolvent, since 1,2,3-propanetriol (glycerol) is one of the main by-products of the biodiesel industry [48], being cheaper than other alcohols such as 1,2,4-butanetriol [49,50].

Three successive dissolution-precipitation cycles were performed with the same solvent:antisolvent system (limonene:1,2,3-propanetriol) under the same conditions (T = 110 °C, S/L ratio = 0.02, 700 rpm, and 30 min), but using a new HDPE sample for each cycle. At the end of each cycle, the recovered HDPE was collected, the pigment was vacuum-filtered, and to the solvent:antisolvent mixture recovered after the precipitation dissolution cycle, we added 7 mL of ethyl acetate, since 1,2,3-propanetriol is not miscible in this solvent, but limonene is, thus the formation of a two-phase liquid-liquid system (LLE). The lower phase of the LLE was composed of 1,2,3-propanetriol, while the upper phase was composed of limonene and ethyl acetate. After phase separation, the lower phase was reused directly, whereas the upper phase was subjected to evaporation at reduced pressure to remove the ethyl acetate through evaporation and recover the limonene from reusing it again in the pretreatment process. A total of three cycles were performed: a first extraction cycle and two more cycles where the solvents recovered from the previous cycle were reused. The results obtained are shown in Figure S12 and Table S8. The recyclability studies demonstrated that it is possible to recover about 60% (*w*/*w*) of the 1,2,3-propanetriol (antisolvent) and about 40% (*w*/*w*) of the limonene (solvent) for the three cycles, with the solvent recovery percentage for both solvents remaining approximately constant over the cycles. Thus, a fresh feed of solvent and antisolvent is required for each cycle since the solvents were not fully recovered during the recovery steps. In addition, the recovery of HDPE after each cycle was almost total, about 90% (*w*/*w*), which can be considered an effective result. Furthermore, the percentage of pigment removed from the recovered HDPE samples after each cycle remained constant, removing 100% of the pigment in the three cycles. Thus, the use of the recovered solvents (limonene and 1,2,3-propanetriol) over the cycles of recyclability did not affect the efficiency of the process, demonstrating the feasibility of the process. 

**Figure 6:**
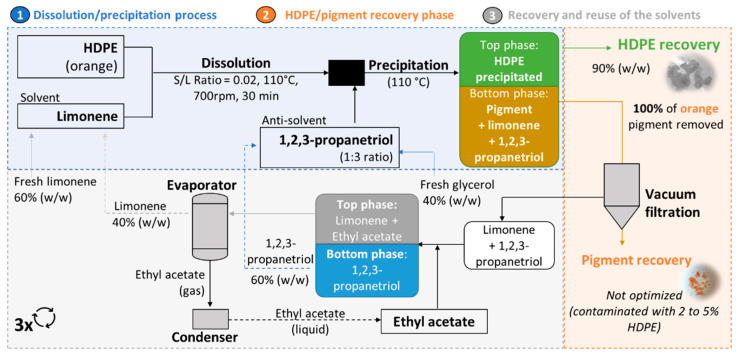
Schematic representation of the developed pretreatment process to remove orange pigment from HDPE using the dissolution-precipitation technique with a limonene:1,2,3-propanetriol system.

In summary, the proposed process is divided into three phases: (1) dissolution-precipitation, (2) HDPE and pigment recovery, and (3) recovery and reuse. This process is schematically represented in Figure 6.

## 3. Materials and Methods

### 3.1. Materials

Two post-consumer HDPE packages were used in this work, one blue and one orange. Before the pretreatment, the lids, labels, and glue were removed from packages, and they were washed with detergent and water to remove surface impurities, and then dried. Next, the packages were cut into small pieces approximately of 0.5 × 0.5 cm.

The chemical compounds used in this work are summarized in Table 3. The water used in this work was ultrapure water, distilled twice, passed through a reverse osmosis system and treated with a Milli-Q Integral 10 (Merck, Darmstadt, Germany) water purification device.

### 3.2. Colorants Identification

#### 3.2.1. Optical Microscopy (OM)

OM was used to identify the color and distribution of the colorants in the polymeric matrixes. Images of samples were acquired using an OLYMPUS BX51 optical microscope (Hamburg, Germany) with an IDS digital camera and uEye IDS software.

#### 3.2.2. Energy Dispersive X-ray Fluorescence Microspectroscopy (μ-EDXRF)

μ-EDXRF analyses were performed on an ArtTAX spectrometer (Intax GmbH, Bruker, Berlin, Germany), equipped with a molybdenum (Mo) anode and an Xflash detector refrigerated by the Peltier effect (Sidrift). The primary X-ray beam was focused to a diameter of 70 μm by means of a polycapillary X-ray mini-lens. The characteristic X-rays emitted by the sample were detected by a silicon drift electro-thermally cooled detector with a resolution of 160 eV at Mn-Kα. The experimental parameters used were: 40 kV voltage, 600 μA intensity, and 360 s of acquisition time. Measurements were carried out in a helium atmosphere when necessary to detect lower elements such as aluminum (Al).

#### 3.2.3. Raman Microscopy (μ-Raman)

μ-Raman was carried out using a Labram 300 Jobin Yvon spectrometer (HORIBA Jobin Yvon, Villeneuve d’Ascq, France), equipped with a HeNe 17 mW laser operating at 632.8 nm. Spectra were recorded as an extended scan. The laser beam was focused with a 50× Olympus objective lens. The laser power at the surface of the samples was varied with the aid of a set of neutral density filters. Samples were analyzed using a 10–20 s laser exposure time for 10-20 scans. The database used for pigment identifications was the SOP Spectral Library (SOPRANO) [51].

### 3.3. Pretreatment of HDPE

#### 3.3.1. Dissolution-Precipitation

The dissolution-precipitation process was also carried out using Radleys Tech Carousel equipment. The first step was to investigate the best solvent (toluene, xylene, cyclohexane, or D-limonene) to be used in the dissolution of the HDPE (blue and orange), and all solvents were used in their pure state. The dissolution studies were carried out with an S/L ratio of 0.02 and a rotation of 700 rpm. The remaining dissolutions conditions were different for each solvent, as described below. The dissolution temperature for each of the solvents was different (Table 1), as discussed in Section 2.2.1. The time of dissolution was 30 min, except for the solvent cyclohexane, for which 420 min was applied.

After finding the solvents suitable for HDPE dissolution (limonene as an alternative solvent and toluene as a comparison), a preliminary study was performed to verify if limonene under toluene conditions (T = 110 °C, 700 rpm for 30 min) has the ability to dissolve HDPE, since it would be of interest to use the same operating conditions for both solvents. After verifying the dissolution of HDPE with limonene under the new conditions, the pre-treatment was started to remove the colorants from the HDPE packaging. The first step was to dissolve the plastics (blue and orange) using two solvents, limonene and toluene, under the previously defined conditions (T = 110 °C, S/L ratio of 0.02, 700 rpm, for 30 min). Then, the alcohols listed in Table 3 were added as antisolvents at different ratios of solvent:antisolvent (1:residual, 1:1, and 1:3) in order to precipitate the polymer. The antisolvent was slowly added drop by drop. After precipitation, the precipitated polymer was removed from the solution and dried in the vacuum line for 48 h in order to remove the solvent and antisolvent residues that remained in the sample. Next, the remaining solution, i.e., the mixture of solvent, antisolvent, and pigment, was vacuum-filtered in order to recover the precipitated/suspended pigment in the solvent mixture. Each dissolution-precipitation cycle was repeated at least three times, which allowed the determination of the average percentage of colorant removed and its standard deviation.

#### 3.3.2. Quantification of Removed Pigment

A Bruker MultiRAM system with a LN-Ge Diode laser at an output of 350 mW, incorporating a liquid nitrogen cooling system, was used for the acquisition of the spectra. The spectra of the samples were obtained through OPUS software and an average of 200 scans was used in the range between 50 and 4000 cm^−1^, with a spectrum resolution of 4 cm^−1^. However, for the recovered samples from the blue HDPE using toluene as the solvent, it was necessary to decrease the laser potency to 10 mW, since under previous operating conditions, the spectra showed large amounts of noise/interference, preventing the detection of a signal during the analysis. 

The spectra of pure HDPE, blue HDPE, orange HDPE, and the respective pigments recovered by FT-Raman were determined to quantify the removed pigment. These spectra were normalized using OriginPro 9 software in relation to the pure HDPE sample, and the most intense band of the spectrum (2912 cm^−1^) was used as a reference. In the spectra obtained (Figure S13), the peak of the blue pigment was located at 1530 cm^−1^ and the peak of the orange pigment was located at 463 cm^−1^. After the identification of the peaks of interest, the spectra of all recovered samples were plotted, and the percentage of pigment removed (% Pigment removed) was determined by Equation (1), through the area of the pigment peak of the recovered samples (A_Pigment in recovered HDPE_) in relation to the area of the pigment peak in the colored HDPE samples before the extraction (A_Pigment colored HDPE_). The areas were calculated using the PeakFit program. A percentage of pigment removal inferior to 100% means that the pigment was not completely removed from the polymer matrix, whereas values equal to 100% indicate that the pigment was completely removed from the polymer matrix.

(1) $$\%\;\mathrm{Pigment}\;\mathrm{removed}=\left(1-\frac{A_{\mathrm{Pigment}\;\mathrm{in}\;\mathrm{recovered}\;\mathrm{HDPE}}}{A_{\mathrm{Pigment}\;\mathrm{in}\;\mathrm{colored}\;\mathrm{HDPE}\;}}\right)\times 100$$

### 3.4. Chemical Characterization of the Polymer before and after Pretreatment

#### 3.4.1. Fourier Transform Infrared Spectroscopy (FT-IR)

A Perkin Elmer Spectrum BX system was used with Spectra software for obtaining the spectra. The samples’ spectra were obtained using an average of 32 scans in the range of 500 to 4000 cm^−1^, with a spectrum resolution of 4 cm^−1^, and air was used as the background. With this technique, all recovered samples after the dissolution-precipitation process were analyzed, and the pure HDPE, blue HDPE, and orange HDPE samples were analyzed for comparison with the recovered HDPE samples.

#### 3.4.2. Thermogravimetric Analysis (TGA) and Differential Scanning Calorimetry (DSC)

For TGA and DSC was used a Setaram, Setsys EV 1750 model and TG-DSC 1500 module (Caluire, France). Samples ranging from 2.5 to 8 mg were weighed and heated from 20 to 600 °C at a heating rate of 10 °C/min under a nitrogen atmosphere. The nitrogen flow rate in the TGA cell was approximately 50 mL/min. A baseline was also performed under the same analysis conditions with empty cuvettes to eliminate the gas’s effect on the cuvettes. The calibration of the equipment was performed by melting 4 standards (indium, lead, aluminum, and gold) at 3 different heating speeds (5, 10, and 15 °C/min). With this technique, the recovered samples with percentages of pigment removal equal to or higher than 80% after the dissolution-precipitation process were analyzed. Pure HDPE, blue HDPE, and orange HDPE samples were also analyzed for comparison with the recovered HDPE samples.

### 3.5. Recyclability of Solvents

For the development of a more sustainable extraction process, the recovery and reuse of solvents after the dissolution-precipitation process were evaluated to identify the most promising system (limonene:1,2,3-propanetriol in the extraction of orange pigment from HPDE). The solvents were recovered through dilution in ethyl acetate, forming two phases: an upper phase rich in limonene and ethyl acetate and a bottom phase rich in 1,2,3-propanetriol. Then, the upper phase was evaporated at reduced pressure to recover limonene, which was then used again in another pigment extraction cycle. A total of three cycles (1 extraction cycle and 2 cycles of solvent recycling) were performed.

## 4. Conclusions

Biosolvents derived from renewable sources were shown to be efficient in the removal of colorants from plastic packaging waste by solvent extraction (dissolution-precipitation). Initially, the type of colorants present in the HDPE packaging was identified, showing that both plastics had just one pigment each. Afterward, several solvents were evaluated for HDPE dissolution, with limonene being found to be the most promising. Then, a wide range of alcohols (mono-, di-, and tri-alcohols, with alkyl chains of up to six carbons) was evaluated as antisolvents in different solvent:antisolvent ratios (1:residual, 1:1, and 1:3) to maximize the yield of the precipitated polymer, with 1:3 being the most effective ratio. Notably, toluene (a conventional organic solvent) was also used as a dissolution solvent for comparative purposes. We found that the removal of pigments was more efficient when using limonene as solvent for the plastic dissolution and alcohols with an intermediate alkyl chain and multiple OH groups as the antisolvent. The pigment removal was most effective for the tri-alcohols (1,2,3-propanetriol and 1,2,4-butanetriol), removing up to 94% and 100% of the blue and orange pigments, respectively, thus showing the potential of these biosolvents to remove the additives from plastic waste, which could be used in the development of a more sustainable plastic waste recycling process. Then, we found that the chemical structure of the polymer did not change significantly after pretreatment, despite, in some samples, the alcohol used in the polymer precipitation partly remaining in the polymer matrix. This did not seem, however, to have affected the structural composition of the polymer. Moreover, the thermal properties of the recovered HDPE, such as decomposition temperature, melting temperature, and heat of fusion, showed similar values to the respective reference polymers. Finally, three cycles of dissolution-precipitation with the reuse of the most promising system (limonene/1,2,3-propanetriol) was performed, to ensure the economic viability and sustainability of the developed pretreatment, showing that the recyclability of the solvents is feasible, since the percentage of pigment removed from the recovered HDPE remained at 100% after the three cycles. In summary, a sustainable pretreatment based on the use of renewable biosolvents (limonene and 1,2,3-propanetriol (glycerol)) was proposed to remove the colorants present in HDPE, without affecting the polymer’s properties. The objective of this pretreatment is to be the first step in facilitating polymer recycling by depolymerization, allowing an increase in the quality of recycled polymers, generating economic value for the waste and new opportunities in plastic recycling.

## Figures and Tables

**Table 1:** A photographic summary of the results obtained after the blue and orange HDPE dissolution process using different (bio)solvents. Fixed dissolution conditions: 0.05 g of HDPE was dissolved in 2.5 mL of solvent (S/L ratio of 0.02) and a rotation speed of 700 rpm. T, temperature; t, time.

Solvent (Dissolution Conditions)	HDPE	
	Blue Pigment	Orange Pigment
Toluene (T = 110 °C; t = 30 min) 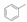	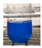	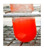
D-limonene (T = 140 °C; t = 30 min) 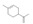	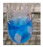	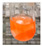
Xylene (T = 140 °C; t = 30 min) 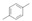	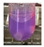	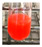
Cyclohexane (T = 80 °C; t = 420 min) 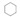	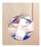	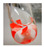

**Table 2:** Melting temperature (T_m_), heat of fusion (∆H_f_), and degree of crystallinity (DC) for the recovered samples with at least 80% pigment removal.

Recovered HDPE	Toluene (Orange HDPE)			Limonene (Blue HDPE)			Limonene (Orange HDPE)		
	T_m_ (°C)	∆H_f_ (J/g)	DC (%)	T_m_ (°C)	∆H_f_ (J/g)	DC (%)	T_m_ (°C)	∆H_f_ (J/g)	DC (%)
1,2-Ethanediol	---	---	---	---	---	---	137.1	226.6	77.3
1,2-Propanediol	---	---	---	---	---	---	134.2	213.9	73.0
1,3-Propanediol	---	---	---	---	---	---	135.9	267.6	91.3
1,4-Butanediol	136.9	215.2	73.4	---	---	---	---	---	---
1,2,3-Propanetriol	133.3	260.7	89.0	133.7	276.6	94.4	135.8	212.2	72.4
1,2,4-Butanetriol	135.4	194.5	66.4	133.6	220.6	75.3	137.2	249.7	85.2

**Table 3:** List of substances used in this work, including the supplier, purity (wt %), and CAS number.

Compound	Supplier	Purity	CAS Number
*Solvent*			
Toluene	Fisher Scientific (Geel Belgium)	99.8	108-88-3
Xylene	JMGS (Odivelas, Portugal)	96.0	1330-20-7
Cyclohexane	Sigma-Aldrich (Steinheim, Germany)	≥99.7	110-82-7
D-limonene	Sigma-Aldrich (Steinheim, Germany	97.0	5989-27-5
*Antisolvent*			
Methanol	Fisher Scientific (Geel Belgium)	99.0	67-56-1
Ethanol	Fisher Scientific (Geel Belgium)	99.0	64-17-5
1-Propanol	Carlo Erba (Val de Reuil, France)	---	71-23-8
2-Propanol	Fisher Scientific (Geel Belgium)	>99.0	67-63-0
1-Butanol	Prolabo (Geel Belgium)	99.5	71-36-3
2-Butanol	Prolabo(Geel Belgium)	100.0	78-92-2
1-Pentanol	Sigma-Aldrich (Steinheim, Germany	>99.0	71-41-0
1-Hexanol	Alfa Aesar, (Kandel, Germany)	99.0	111-27-3
1,2-Ethanediol	Fisher Scientific (Geel Belgium)	>99.0	107-21-1
1,2-Propanediol	Sigma-Aldrich (Steinheim, Germany)	99.5	57-55-6
1,3-Propanediol	Sigma-Aldrich (Steinheim, Germany)	98.0	504-63-2
1,2-Butanediol	Sigma-Aldrich (Steinheim, Germany)	98.0	584-03-2
1,3-Butanediol	Sigma-Aldrich (Steinheim, Germany)	99.5	107-88-0
1,4-Butanediol	Alfa Aesar (Kandel, Germany)	99.0	110-63-4
1,2-Pentanediol	TCI (Zwijndrecht, Belgium)	>98.0	5343-92-0
1,5-Pentanediol	Alfa Aesar, (Kandel, Germany)	97.0	111-29-5
1,2-Hexanediol	Alfa Aesar (Kandel, Germany)	97.0	6920-22-5
1,6-Hexanediol	Acros Organics (Geel Belgium)	97.0	629-11-8
1,2,3-Propanetriol	Fisher Chemical (Geel Belgium)	99.9	56-81-5
1,2,4-Butanetrioll	Sigma-Aldrich (Steinheim, Germany	98.0	42890-76-6
*Others*			
Ethyl acetate	Fisher Scientific (Geel Belgium)	≥99.5%	141-78-6

## Data Availability

Not applicable.

## Supplementary Materials

The following are available online, Figure S1. Energy dispersive X-ray fluorescence spectra; Figure S2. Raman spectra; Figure S3. Energy dispersive X-ray fluorescence spectra; Figure S4. Raman spectra; Figure S5. Vibrational spectra obtained by FT-IR of recovered HDPE samples under the pretreatment conditions. Blue HDPE treated with toluene as solvent: (A) mono-, (B) di-, and (C) tri-alcohols; Figure S6. Vibrational spectra obtained by FT-IR of recovered HDPE samples under the pretreatment conditions. Orange HDPE treated with toluene as solvent: (A) mono-, (B) di-, and (C) tri-alcohols; Figure S7. Vibrational spectra obtained by FT-IR of recovered HDPE samples under the pretreatment conditions. Blue HDPE treated with limonene as solvent: (A) mono-, (B) di-, and (C) tri-alcohols; Figure S8. Vibrational spectra obtained by FT-IR of recovered HDPE samples under the pretreatment conditions. Orange HDPE treated with limonene as solvent: (A) mono-, (B) di-, and (C) tri-alcohols; Figure S9. Vibrational spectra obtained by FT-IR: (black) pure HDPE, (blue) blue HDPE and (orange) orange HDPE; Figure S10. TGA curves of recovered HDPE under different pretreatment conditions: (A) orange HDPE treated with toluene as solvent, and (B) blue HDPE and (C) orange HDPE, both treated with limonene as solvent. The different curves represent the recovered HDPE using the antisolvents: (yellow) 1,2-ethanediol, (pink) 1,2-propanediol, (grey) 1,3-propanediol, (purple)1,4- butanediol, (red) 1,2,3-propanetriol, and (green) 1,2,4-butanetriol. Controls: (black) pure HDPE, (blue) blue HDPE, and (orange) orange HDPE; Figure S11. DSC curves of recovered HDPE under different pretreatment conditions: (A) orange HDPE treated with toluene as solvent, and (B) blue HDPE and (C) orange HDPE, both treated with limonene as solvent. The different curves represent the recovered HDPE using the following antisolvents: (yellow) 1,2-ethanediol, (pink) 1,2-propanediol, (grey) 1,3-propanediol, (purple)1,4- butanediol, (red) 1,2,3-propanetriol, and (green) 1,2,4-butanetriol. Controls: (black) pure HDPE, (blue) blue HDPE, and (orange) orange HDPE; Figure S12. Percentage recovery of the (blue) limonene, (yellow) 1,2,3-propanetriol, (green) HDPE, and the (orange) pigment removed from the HDPE samples for each recyclability cycle using the dissolution-precipitation conditions: T = 110 °C and S/L ratio = 0.02; Figure S13. Representation of the vibrational spectra by FT-RAMAM of the samples related to: (A) blue HDPE and (B) orange HDPE (black) pure HDPE, (blue) blue HDPE, (dark green) orange HDPE, (light blue) recovered blue pigment, and (orange) recovered orange pigment; Table S1. A photographic summary of the results obtained after the polymer precipitation process for the different solvent:antisolvent ratios (1:residual, 1:1, and 1:3), here using 1,2-ethanediol as antisolvent; Table S2. Percentage of pigment removed from blue and orange HDPE. Polymer matrix dissolved in toluene or limonene using a solvent:antisolvent ratio of 1:3. Table S3. Identification of characteristic bands present (green check mark) or not present (red check mark) in recovered samples of blue HDPE treated with toluene as solvent; Table S4. Identification of characteristic bands present (green check mark) or not present (red check mark) in recovered samples of orange HDPE treated with toluene as solvent; Table S5. Identification of characteristic bands present (green check mark) or not present (red check mark) in recovered samples of blue HDPE treated with limonene as solvent; Table S6. Identification of characteristic bands present (green check mark) or not present (red check mark) in recovered samples of orange HDPE treated with limonene as solvent; Table S7. Temperatures of maximum decomposition rate (T_max_) and temperature at which 1% of the sample decomposed (T_onset_), only for the recovered samples with at least 80% pigment removal; Table S8. Percentage recovery of the limonene, 1,2,3-propanetriol, HDPE, and the pigment from the HDPE samples for each recyclability cycle using the dissolution-precipitation conditions: T = 110 °C and S/L ratio = 0.02.

## Sample Availability

Samples of the compounds are not available from the authors.
