# Supplementary material for: Pretreatment of Plastic Waste: Removal of Colorants from HDPE Using Biosolvents
Source: Molecules. 2021 Dec 24;27(1):98. doi: 10.3390/molecules27010098 (PMC8746977; doi:10.3390/molecules27010098)
Supplement: Supplementary file 1 [file molecules-27-00098-s001.zip › molecules-1511930-supplementary.pdf]

Supplementary Material

Pretreatment of Plastic Waste: Removal of Colorants from HDPE Using Biosolvents

Ana M. Ferreira [1,†,*], Isa Sucena[1,†], Vanessa Otero [2,3], Eva Mariasole Angelin [2,4], Maria João Melo [2] and João A. P. Coutinho [1]

[1] CICECO—Aveiro Institute of Materials, Department of Chemistry, University of Aveiro, 3810-193 Aveiro, Portugal; isasucena@ua.pt (I.S.); jcoutinho@ua.pt (J.A.P.C.)

[2] LAQV-REQUIMTE, Department of Conservation and Restoration, Faculdade de Ciências e Tecnologia, Universidade NOVA de Lisboa, 2829-516 Monte da Caparica, Portugal; van_otero@fct.unl.pt (V.O.); e.angelin@campus.fct.unl.pt (E.M.A.); mjm@fct.unl.pt (M.J.M.)

[3] VICARTE, Department of Conservation and Restoration, Faculdade de Ciências e Tecnologia,
Universidade NOVA de Lisboa, 2829-516 Monte da Caparica, Portugal

[4] Conservation Science Department, Deutsches Museum, Museumsinsel 1, 80538 Munich, Germany

* Correspondence: ana.conceicao@ua.pt

† These authors contributed equally to this work.

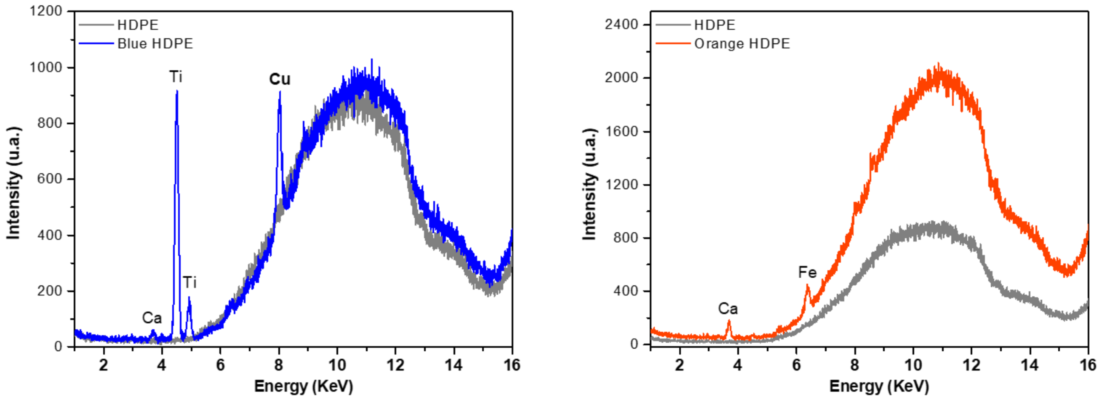

Figure S1. Energy dispersive X-ray fluorescence spectra.

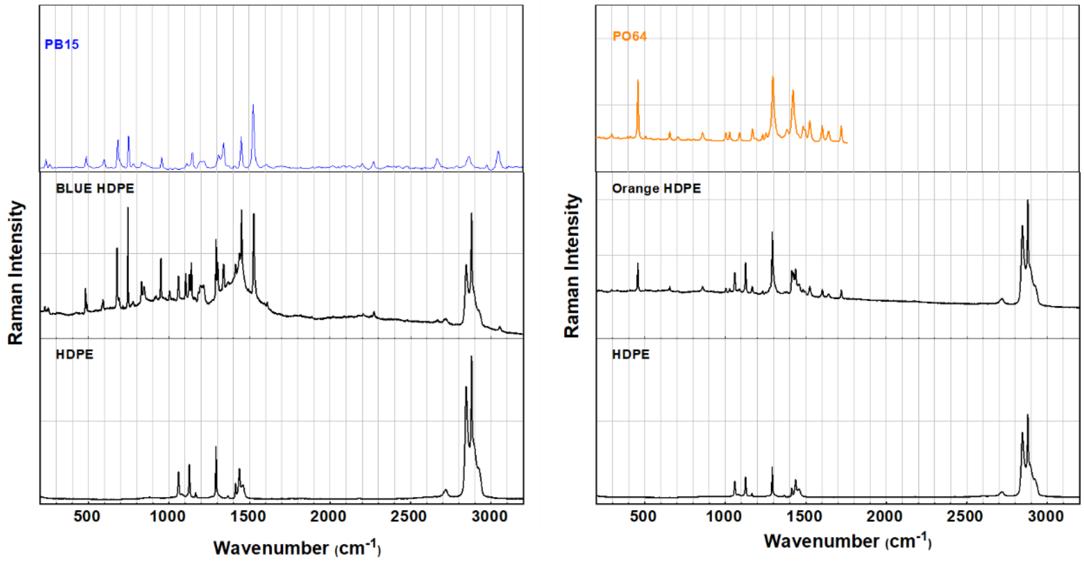

Figure S2. Raman spectra.

Table S1. A photographic summary of the results obtained after the polymer precipitation process for the different solvent:antisolvent ratios (1:residual, 1:1 and 1:3), here using 1,2-ethanediol as antisolvent.

**Solvent**	**Solvent:antisolvent ratio**		
	1:Residual	1:1	1:3
**Toluene**	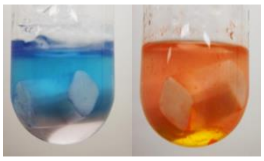	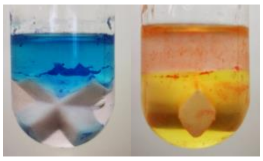	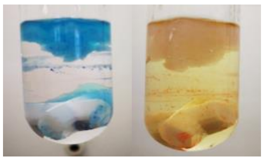
**Limonene**	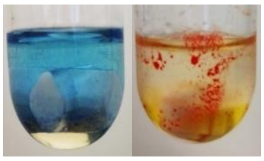	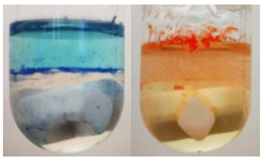	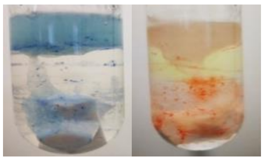

Table S2. Percentage of pigment removed from blue and orange HDPE. Polymer matrix dissolved in toluene or limonene using a solvent:antisolvent ratio of 1:3.

Antisolvent	Toluene		Limonene	
	Blue HDPE	Orange HDPE	Blue HDPE	Orange HDPE
**Others mono-alcohols**	0%	0%	0%	0%
**Ethanol**	0%	0%	32.3 ± 2.4%	7.6 ± 2.6%
**1,2-Ethanediol**	0%	29.3 ± 1.4%	52.5 ± 1.1%	83.4 ± 1.9%
**1,2-Propanediol**	0%	36.0 ± 2.9%	36.4 ± 3.2%	97.1 ± 3.9%
**1,3-Propanediol**	0%	37.3 ± 3.4%	74.7 ± 0.9%	98.4 ± 1.8%
**1,2-Butanediol**	0%	0%	0%	0%
**1,3-Butanediol**	0%	0%	38.2 ± 1.1%	46.3 ± 1.4%
**1,4-Butanediol**	0%	86.3 ± 2.9%	91.0 ± 3.1%	91.9 ± 3.4%
**1,2-Pentanediol**	0%	0%	0%	0%
**1,5-Pentanediol**	0%	0%	32.2 ± 2.8%	37.1 ± 3.7%
**1,2-Hexanediol**	0%	0%	0%	0%
**1,6-Hexanediol**	0%	0%	23.9 ± 3.0%	33.8 ± 3.1%
**1,2,3-Propanetriol**	0%	100%	94.4 ± 2.6%	100%
**1,2,4-Butanediol**	0%	100%	92.9 ± 1.8%	100%

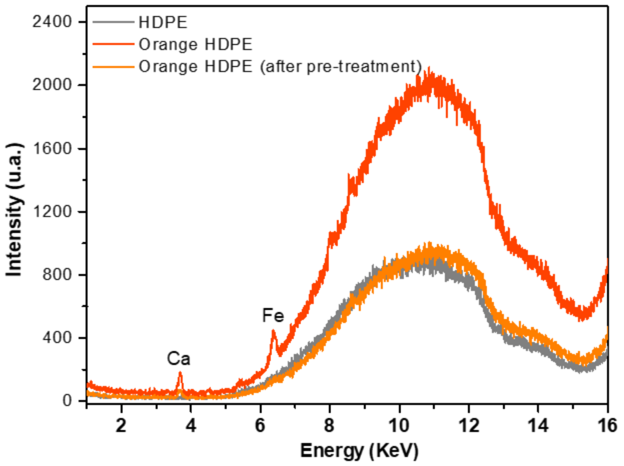

Figure S3. Energy dispersive X-ray fluorescence spectra.

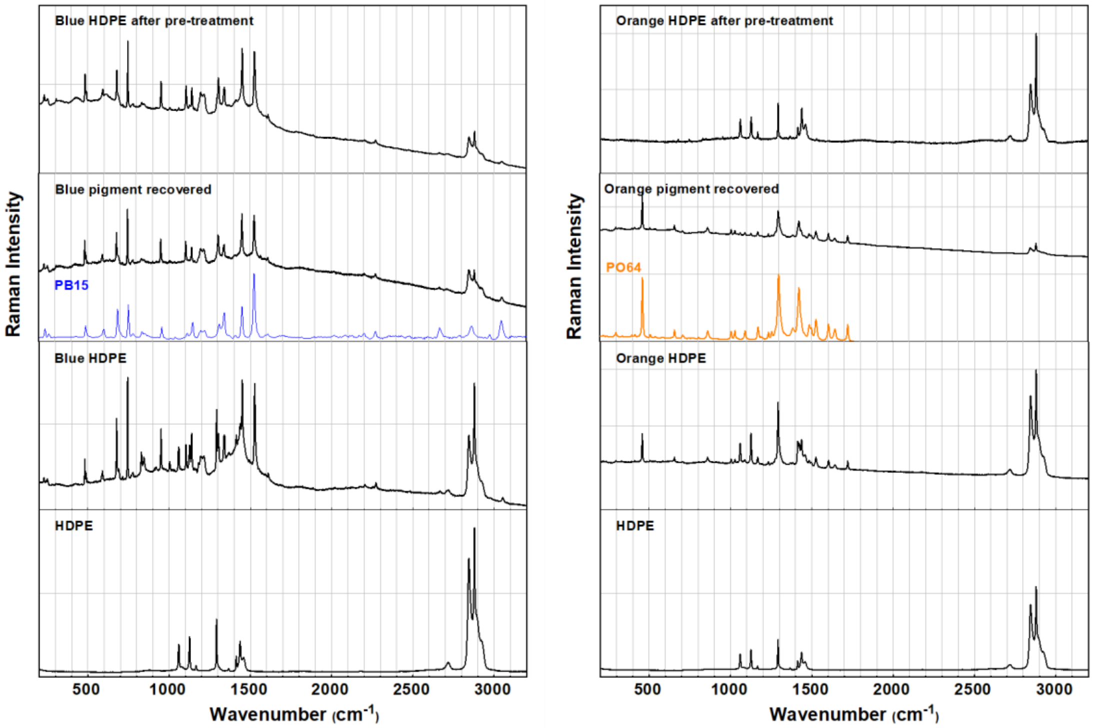

Figure S4. Raman spectra.

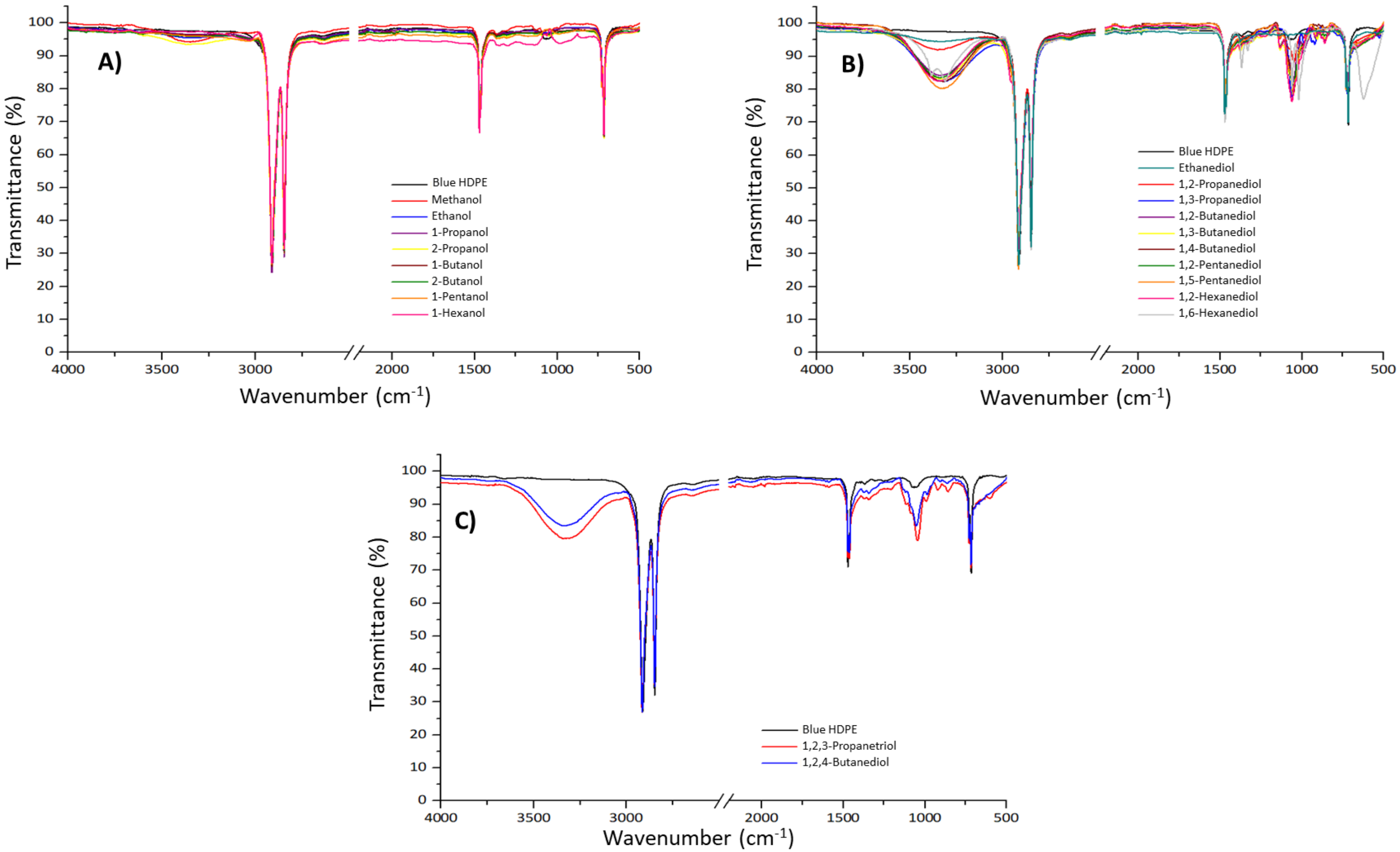

Figure S5. Vibrational spectra obtained by FT-IR of recovered HDPE samples under the pretreatment conditions. Blue HDPE treated with toluene as solvent: (A) mono-, (B) di-, and (C) tri-alcohols.

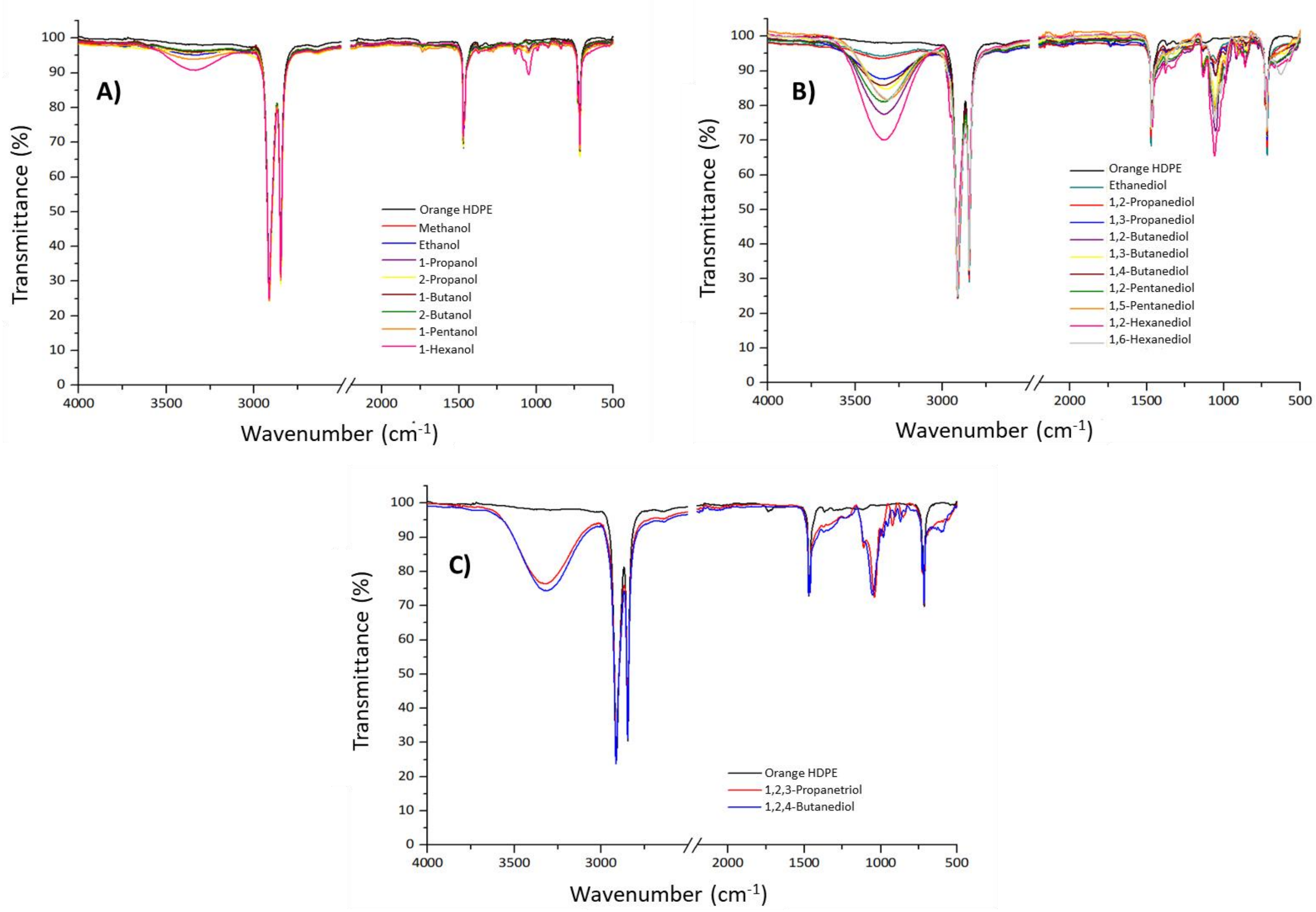

Figure S6. Vibrational spectra obtained by FT-IR of recovered HDPE samples under the pretreatment conditions. Orange HDPE treated with toluene as solvent: (A) mono-, (B) di-, and (C) tri-alcohols.

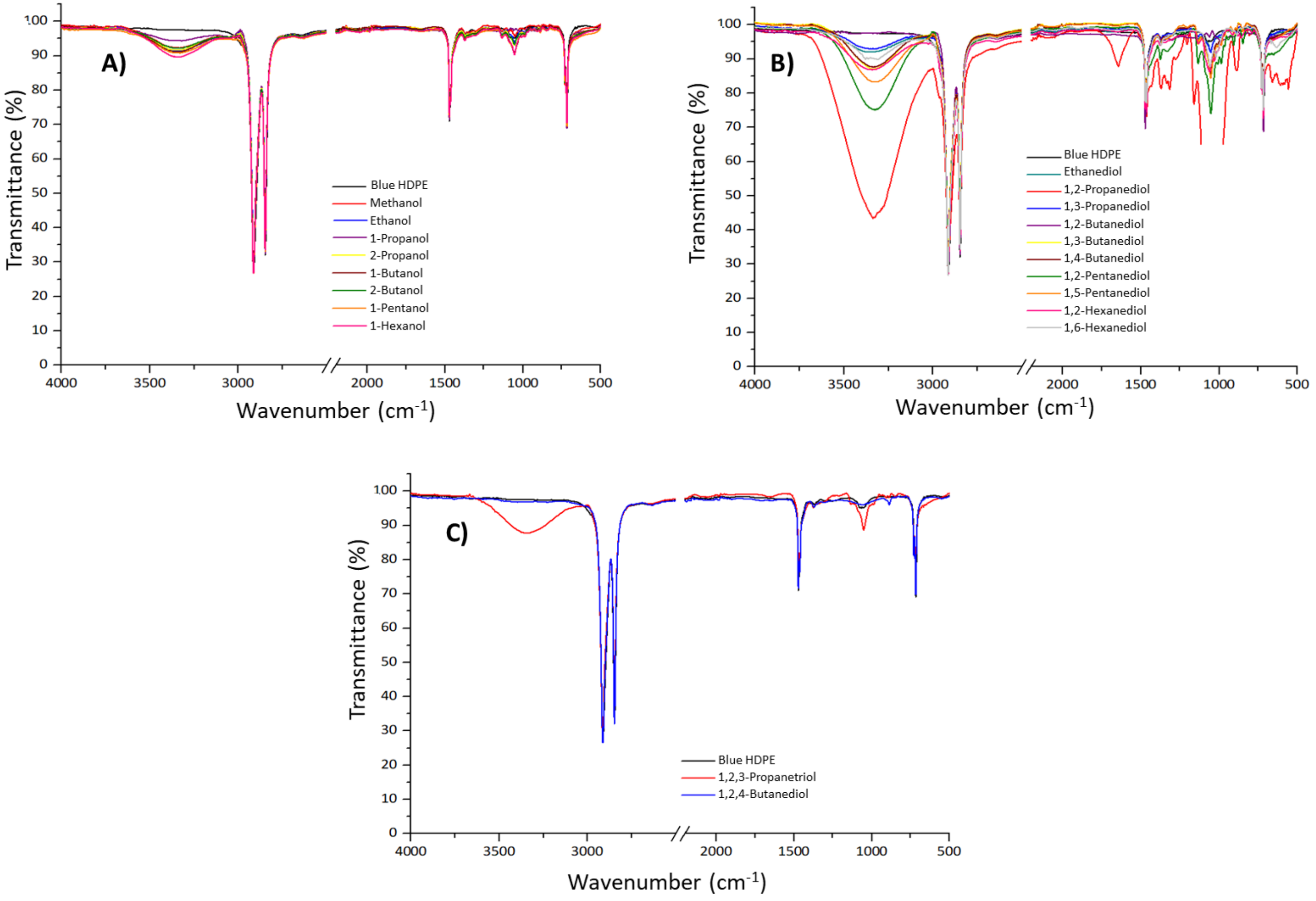

Figure S7. Vibrational spectra obtained by FT-IR of recovered HDPE samples under the pretreatment conditions. Blue HDPE treated with limonene as solvent: (A) mono-, (B) di-, and (C) tri-alcohols.

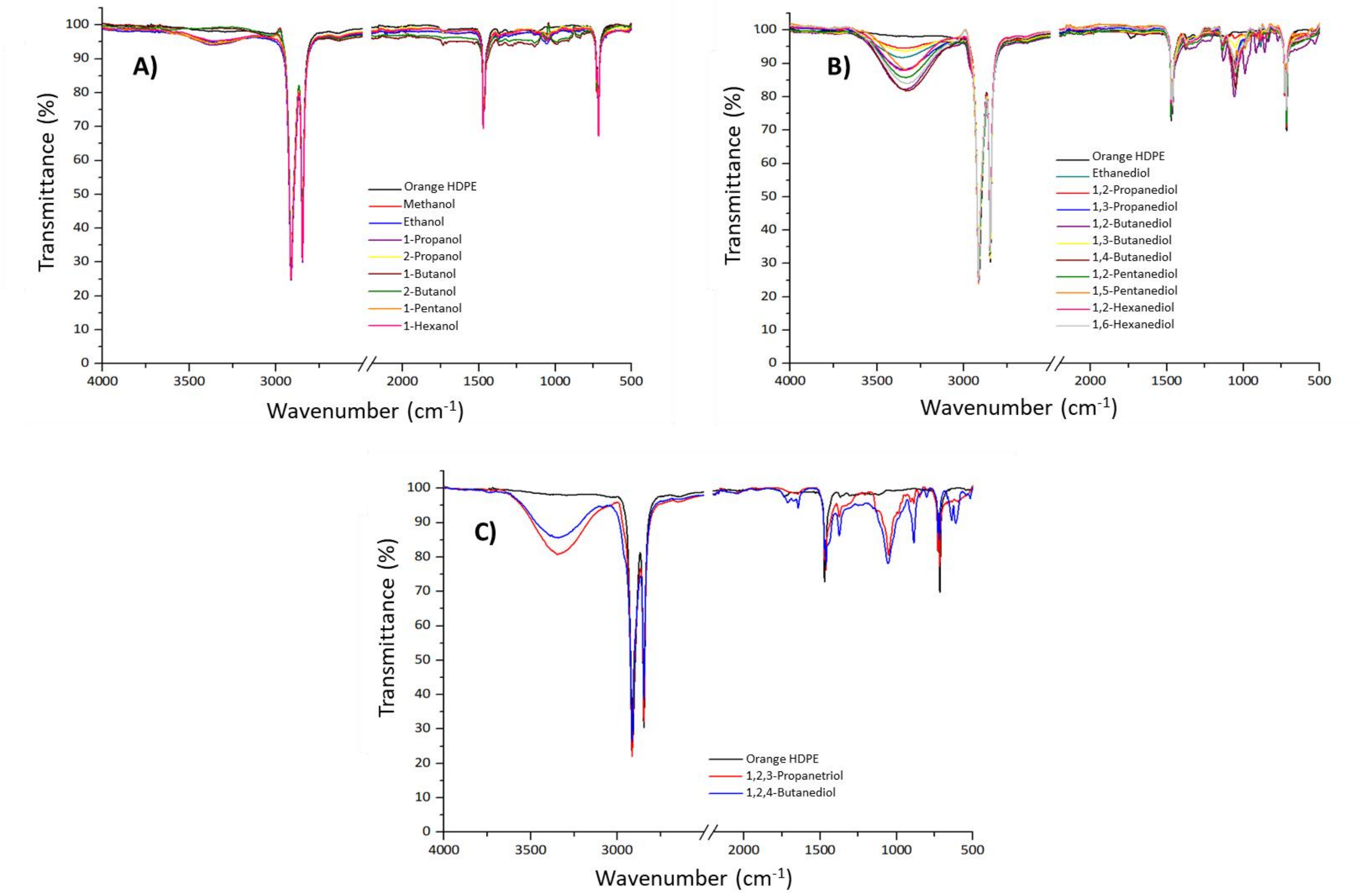

Figure S8. Vibrational spectra obtained by FT-IR of recovered HDPE samples under the pretreatment conditions. Orange HDPE treated with limonene as solvent: (A) mono-, (B) di-, and (C) tri-alcohols.

Table S3. Identification of characteristic bands present (green check mark) or not present (red check mark) in recovered samples of blue HDPE treated with toluene as solvent.

**Blue HDPE**	**Characteristic bands**						
	CH	CH_2	CH_3	CH_2 *rocking*	Blue pigment	OH	CO
**Others mono-alcohols**	✓	✓	✓	✓	---	✗	✗
**Ethanol**	✓	✓	✓	✓	---	✓	✓
**1,2-Ethanediol**	✓	✓	✓	✓	---	✓	✓
**1,2-Propanediol**	✓	✓	✓	✓	---	✓	✓
**1,3-Propanediol**	✓	✓	✓	✓	---	✓	✓
**1,2-Butanediol**	✓	✓	✓	✓	---	✓	✓
**1,3-Butanediol**	✓	✓	✓	✓	---	✓	✓
**1,4-Butanediol**	✓	✓	✓	✓	---	✓	✓
**1,2-Pentanediol**	✓	✓	✓	✓	---	✓	✓
**1,5-Pentanediol**	✓	✓	✓	✓	---	✓	✓
**1,2-Hexanediol**	✓	✓	✓	✓	---	✓	✓
**1,6-Hexanediol**	✓	✓	✓	✓	---	✓	✓
**1,2,3-Propanetriol**	✓	✓	✓	✓	---	✓	✓

Table S4. Identification of characteristic bands present (green check mark) or not present (red check mark) in recovered samples of orange HDPE treated with toluene as solvent.

Orange HDPE	Characteristic bands						
	CH	CH_2	CH_3	CH_2 *rocking*	Orange pigment	OH	CO
**Others mono-alcohols**	✓	✓	✓	✓	✓	✓	✓
**Ethanol**	✓	✓	✓	✓	✓	✓	✓
**1,2-Ethanediol**	✓	✓	✓	✓	✓	✓	✓
**1,2-Propanediol**	✓	✓	✓	✓	✓	✓	✓
**1,3-Propanediol**	✓	✓	✓	✓	✓	✓	✓
**1,2-Butanediol**	✓	✓	✓	✓	✓	✓	✓
**1,3-Butanediol**	✓	✓	✓	✓	✗	✓	✓
**1,4-Butanediol**	✓	✓	✓	✓	✓	✓	✓
**1,2-Pentanediol**	✓	✓	✓	✓	✓	✓	✓
**1,5-Pentanediol**	✓	✓	✓	✓	✓	✓	✓
**1,2-Hexanediol**	✓	✓	✓	✓	✓	✓	✓
**1,6-Hexanediol**	✓	✓	✓	✓	✗	✓	✓
**1,2,3-Propanetriol**	✓	✓	✓	✓	✗	✓	✓

Table S5. Identification of characteristic bands present (green check mark) or not present (red check mark) in recovered samples of blue HDPE treated with limonene as solvent.

**Blue HDPE**	**Characteristic bands**						
	CH	CH_2	CH_3	CH_2 *rocking*	Blue pigment	OH	CO
**Others mono-alcohols**	✓	✓	✓	✓	---	✓	✓
**Ethanol**	✓	✓	✓	✓	---	✓	✓
**1,2-Ethanediol**	✓	✓	✓	✓	---	✓	✓
**1,2-Propanediol**	✓	✓	✓	✓	---	✓	✓
**1,3-Propanediol**	✓	✓	✓	✓	---	✓	✓
**1,2-Butanediol**	✓	✓	✓	✓	---	✓	✓
**1,3-Butanediol**	✓	✓	✓	✓	---	✓	✓
**1,4-Butanediol**	✓	✓	✓	✓	---	✓	✓
**1,2-Pentanediol**	✓	✓	✓	✓	---	✓	✓
**1,5-Pentanediol**	✓	✓	✓	✓	---	✓	✓
**1,2-Hexanediol**	✓	✓	✓	✓	---	✓	✓
**1,6-Hexanediol**	✓	✓	✓	✓	---	✓	✓
**1,2,3-Propanetriol**	✓	✓	✓	✓	---	✗	✗

Table S6. Identification of characteristic bands present (green check mark) or not present (red check mark) in recovered samples of orange HDPE treated with limonene as solvent.

Orange HDPE	Characteristic bands						
	C–H	CH_2	CH_3	CH_2 *rocking*	Orange pigment	OH	CO
**Others mono-alcohols**	✓	✓	✓	✓	✓	✓	✓
**Ethanol**	✓	✓	✓	✓	✓	✓	✓
**1,2-Ethanediol**	✓	✓	✓	✓	✗	✓	✓
**1,2-Propanediol**	✓	✓	✓	✓	✗	✓	✓
**1,3-Propanediol**	✓	✓	✓	✓	✓	✓	✓
**1,2-Butanediol**	✓	✓	✓	✓	✓	✓	✓
**1,3-Butanediol**	✓	✓	✓	✓	✗	✓	✓
**1,4-Butanediol**	✓	✓	✓	✓	✓	✓	✓
**1,2-Pentanediol**	✓	✓	✓	✓	✓	✓	✓
**1,5-Pentanediol**	✓	✓	✓	✓	✓	✓	✓
**1,2-Hexanediol**	✓	✓	✓	✓	✓	✓	✓
**1,6-Hexanediol**	✓	✓	✓	✓	✗	✓	✓
**1,2,3-Propanetriol**	✓	✓	✓	✓	✗	✓	✓

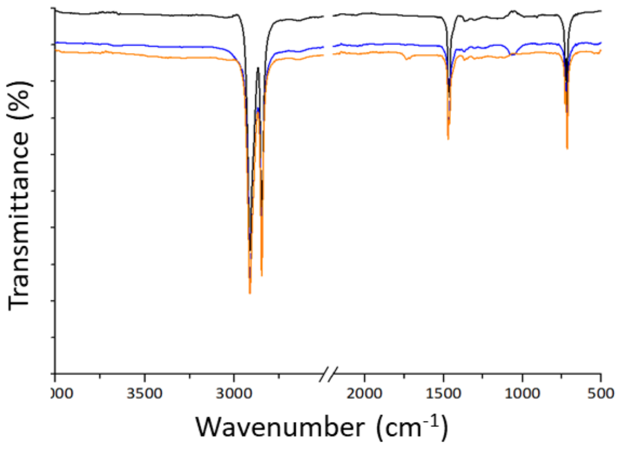

Figure S9. Vibrational spectra obtained by FT-IR: (black) pure HDPE, (blue) blue HDPE and (orange) orange HDPE.

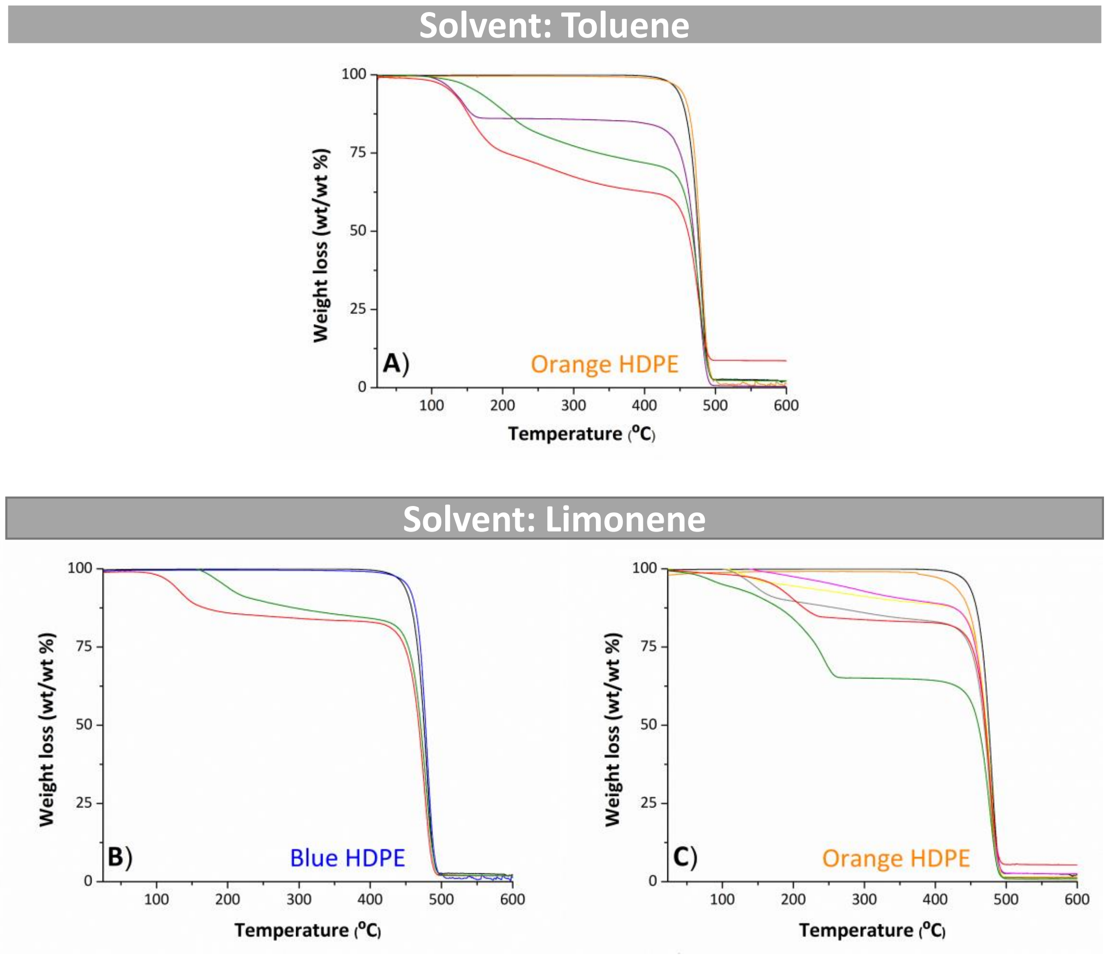

Figure S10. TGA curves of recovered HDPE under different pretreatment conditions: (A) orange HDPE treated with toluene as solvent, and (B) blue HDPE and (C) orange HDPE, both treated with limonene as solvent. The different curves represent the recovered HDPE using the antisolvents: (yellow) 1,2-ethanediol, (pink) 1,2-propanediol, (grey) 1,3-propanediol, (purple)1,4- butanediol, (red) 1,2,3-propanetriol and (green) 1,2,4-butanetriol. Controls: (black) pure HDPE, (blue) blue HDPE (orange) orange HDPE.

Table S7. Temperatures of maximum decomposition rate (T_{max}) and temperature at which 1% the of the sample decomposed (T_{onset}), only for the recovered samples with at least 80% pigment removal.

Recovered HDPE	Toluene (Orange HDPE)		Limonene (Blue HDPE)		Limonene (Orange HDPE)	
	T_{onset} (°C)	T_{max} (°C)	T_{onset} (°C)	T_{max} (°C)	T_{onset} (°C)	T_{max} (°C)
**1,2-Ethanediol**	---	---	---	---	79.4<br>445.6	132.6<br>475.4
**1,2-Propanediol**	---	---	---	---	87.4<br>448.8	122.9<br>475.7
**1,3-Propanediol**	---	---	---	---	81.9<br>446.1	144.6<br>475.3
**1,4-Butanediol**	86.8<br>447.5	145.4<br>475.9	---	---	---	---
**1,2,3-Propanetriol**	96.5<br>449.6	150.9<br>475.3	84.4<br>450.9	132.0<br>478.4	45.5<br>448.2	206.4<br>475.3
**1,2,4-Butanetriol**	129.9<br>444.2	207.7<br>475.1	137.4<br>448.9	196.1<br>478.6	125.9<br>448.0	243.1<br>475.3

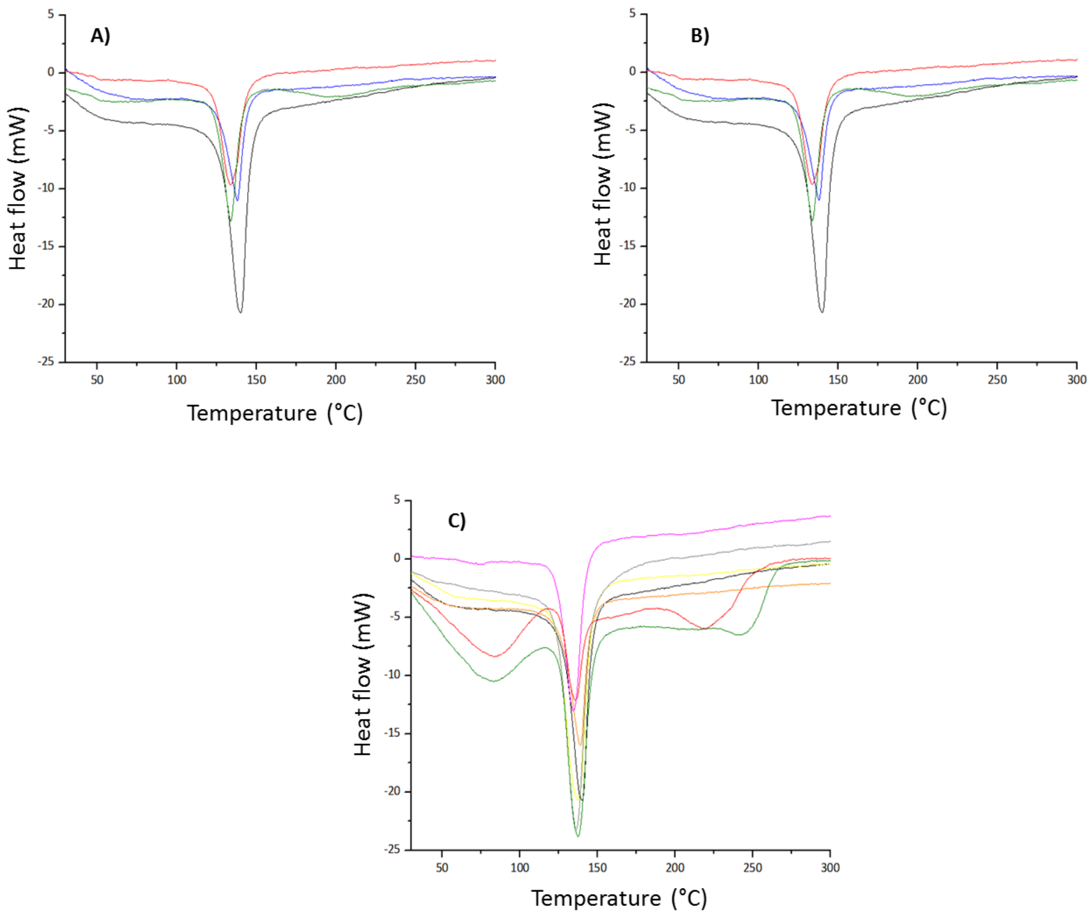

Figure S11. DSC curves of recovered HDPE under different pretreatment conditions. (A) orange HDPE treated with toluene as solvent, and (B) blue HDPE and (C) orange HDPE, both treated with limonene as solvent. The different curves represent the recovered HDPE using the following antisolvents: (yellow) 1,2-ethanediol, (pink) 1,2-propanediol, (grey) 1,3-propanediol, (purple) 1,4- butanediol, (red) 1,2,3-propanetriol and (green) 1,2,4-butanetriol. Controls: (black) pure HDPE, (blue) blue HDPE (orange) orange HDPE.

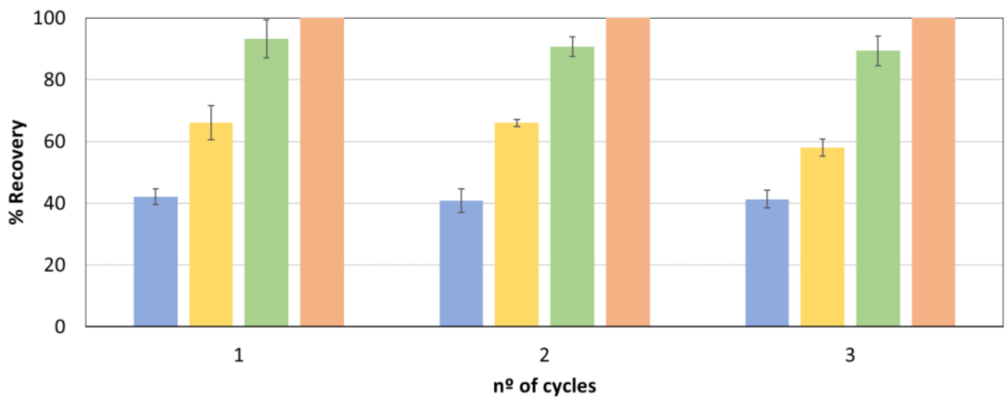

Figure S12. Percentage recovery of the (blue) limonene, (yellow) 1,2,3-propanetriol, (green) HDPE and the (orange) pigment removed from the HDPE samples for each recyclability cycle using the dissolution-precipitation conditions: T = 110°C and S/L ratio = 0.02.

Table S8. Percentage recovery of the limonene, 1,2,3-propanetriol, HDPE and the pigment removed from the HDPE samples for each recyclability cycle using the dissolution-precipitation conditions: T = 110°C and S/L ratio = 0.02.

**Nº of Cycles**	**Solvent (limonene) recovery (%)**	**Solvent (1,2,3-propanetriol) recovery (%)**	**HDPE recovery (%)**	**Pigment removed (%)**
**1**	42.1 ± 2.5	66.1 ± 5.5	93.2 ± 6.1	100
**2**	40.8 ± 3.9	66.0 ± 1.2	90.6 ± 3.2	100
**3**	41.3 ± 2.8	58.0 ± 2.7	89.3 ± 4.7	100

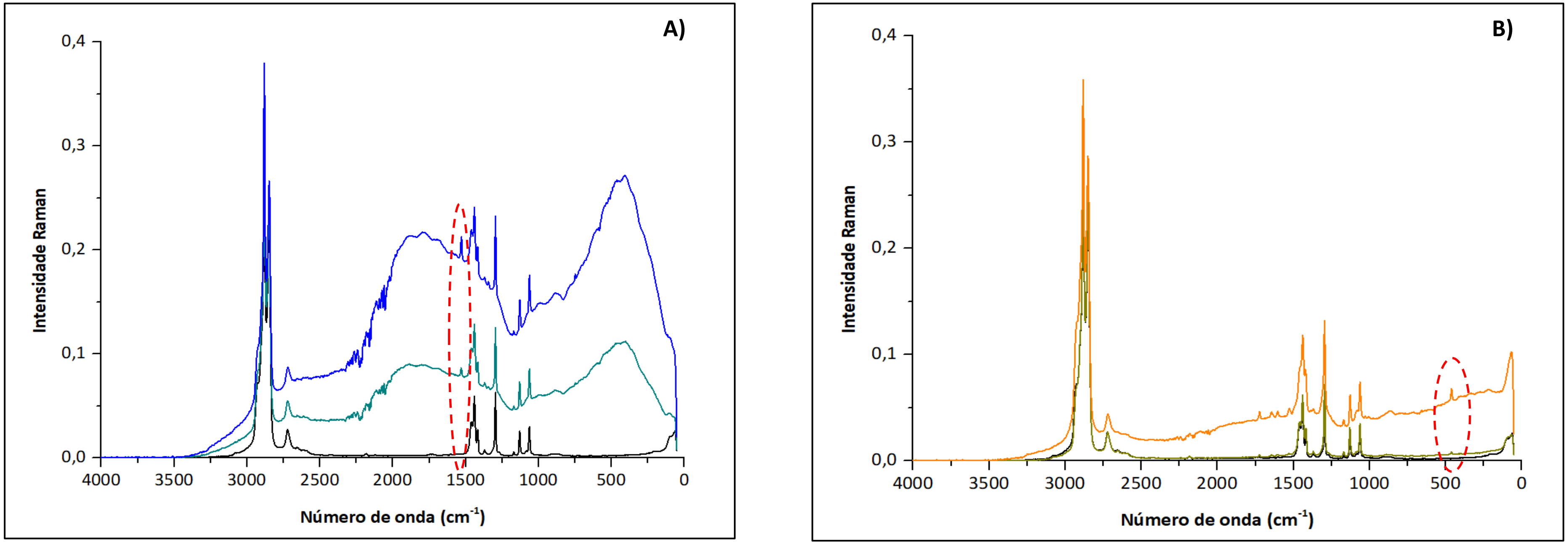

Figure S13. Representation of the vibrational spectra by FT-RAMAM of the samples related to:(A) blue HDPE and (B) orange HDPE. (black) pure HDPE, (blue) blue HDPE, (dark green) orange HDPE, (light blue) recovered blue pigment, (orange) recovered orange pigment.
